# Supplementary material for: Endogenously Expressed Antigens Bind Mammalian RNA via Cationic Domains that Enhance Priming of Effector CD8 T Cells by DNA Vaccination
Source: Mol Ther. 2019 Jan 22;27(3):661–72. doi: 10.1016/j.ymthe.2019.01.011 (PMC6403493; doi:10.1016/j.ymthe.2019.01.011)
Supplement: Document S1. Figures S1–S5 [file mmc1.pdf]

YMTHE, Volume *27*

Supplemental Information

Endogenously Expressed Antigens Bind Mammalian RNA via Cationic Domains that Enhance Priming of Effector CD8 T Cells by DNA Vaccination

Jana Krieger, Petra Riedl, Katja Stifter, Gleyder Roman-Sosa, Thomas Seufferlein, Martin Wagner, and Reinhold Schirmbeck

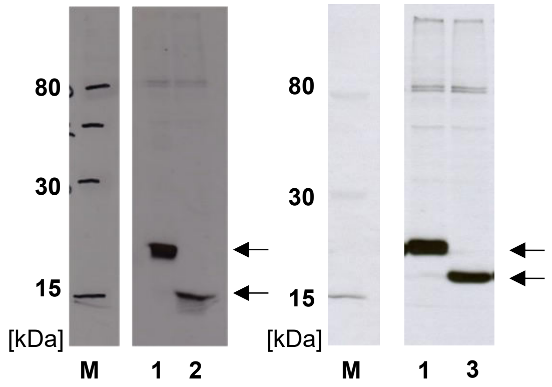

HEK-293 transfected with:

1) pCI/C
2) pCI/C149
3) pCI/C149tat

Supplementary Fig. S1

Expression of HBV-C, HBV-C149 and HBV-C149tat antigens in transiently transfected HEK-293 cells

Expression of the HBV core antigens was determined in two independent experiments. HEK-293 cells were transiently transfected with pCI/C (lanes 1), pCI/C149 (lane 2) or pCI/C149tat DNA (lane 3), labelled with 100µCi ^{35}S-methionine/cysteine at 36h post transfection for 12h and lysed with pH 8.0 lysis buffer (100 mM NaCl, 0.5% NP40 and 100 mM Tris-hydrochloride) supplemented with Protease Inhibitor Cocktail Tablets (cat. no. 11836145001, Roche Applied Science, Penzberg, Germany). Extracts were cleared by centrifugation and precipitated with a polyclonal rabbit anti core serum and protein-A sepharose. Precipitates were processed for SDS-PAGE and subsequent fluorography of the gels. The positions of the respective core antigens are indicated (black arrows). The molecular weight marker (in kDa) is indicated.

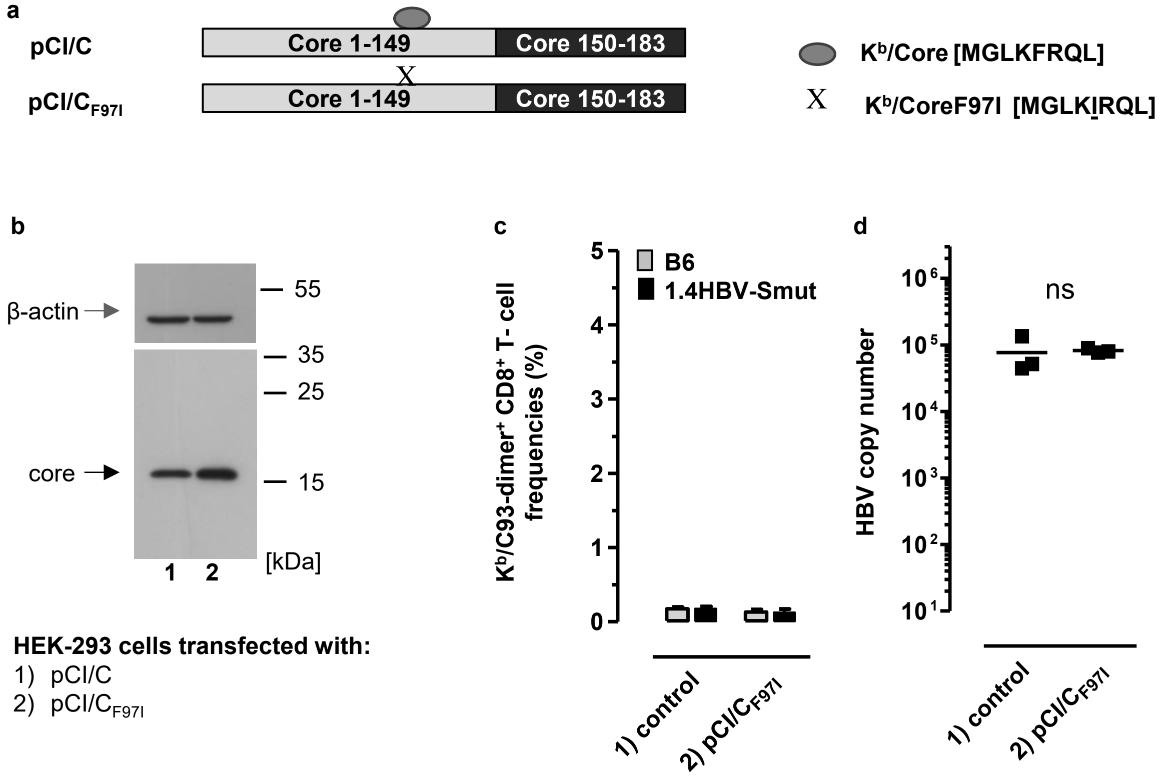

Supplementary Fig. S2

Characterization of the HBV-C antigen harbouring a mutated Kb/C93 epitope

(a) Schematic presentation of HBV-C and HBV-C_{F97I} antigens. The K^b/C93 epitope is given. The HBV-C_{F97I} construct harbors a point mutation at position C97 (exchange of an F to an I) and is indicated by a X. **(b)** Lysates of HEK-293 cells, transiently transfected with the indicated plasmids, were processed for SDS-PAGE followed by Core- and actin-specific western blot analysis. The positions of beta-actin (gray arrow) and HBV core antigens (black arrow) are shown. The molecular weight marker (in kDa) is indicated. **(c)** B6 and 1.4HBV-S^{mut} tg mice were either non-treated (control; groups 1; n=3) or injected with pCI/C_{F97I} (groups 2; n=3) DNA. Twelve days post immunization, K^b/C93-specific dimer$^+$ $CD8^+$ T-cell responses were determined in the liver. The mean % of K^b/C93-specific dimer$^+$ $CD8^+$ T cells in the hepatic $CD8^+$ T-cell populations ($\pm$SD) is shown. **(d)** HBV replication was determined in the liver of non-treated (control) and vaccinated 1.4HBV-S^{mut} tg mice by qRT-PCR as described in the M&M section. The statistical significance of differences between groups 1 and 2 was determined by the unpaired Student's t-test. ns: not significant.

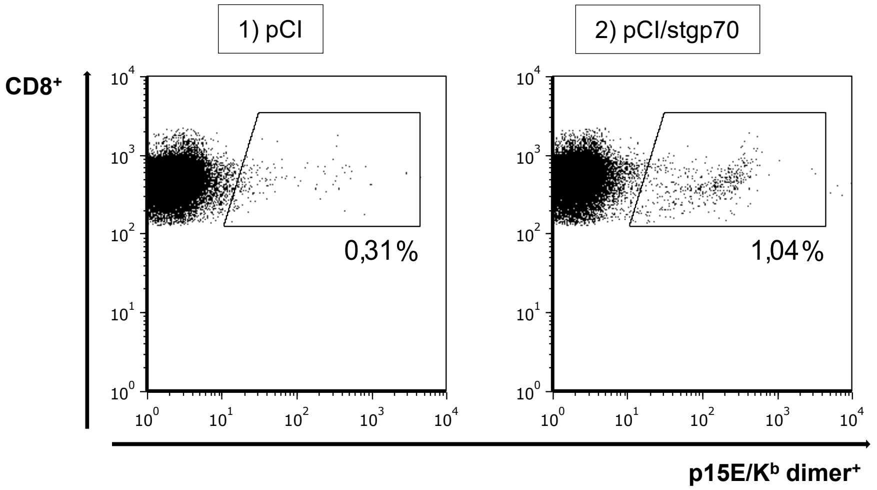

Supplementary Fig. S3

Representative FCM analysis of pCI and pCI/stgp70 vaccinated mice shown in figure 2c.

B6 mice were immunized i.m. with pCI (left panel) and pCI/stgp70 (right panel). Twelve days post immunization, antigen-specific K^b/p15E-dimer$^+$ CD8$^+$ T-cell responses were determined in preparations of spleens by FCM. In this experiment, two out of six pCI/stgp70-immune mice showed priming of K^b/p15E-dimer$^+$ CD8$^+$ T cells. One of the two positive K^b/p15E-dimer$^+$ CD8$^+$ T-cell populations are shown in dot blot analyses.

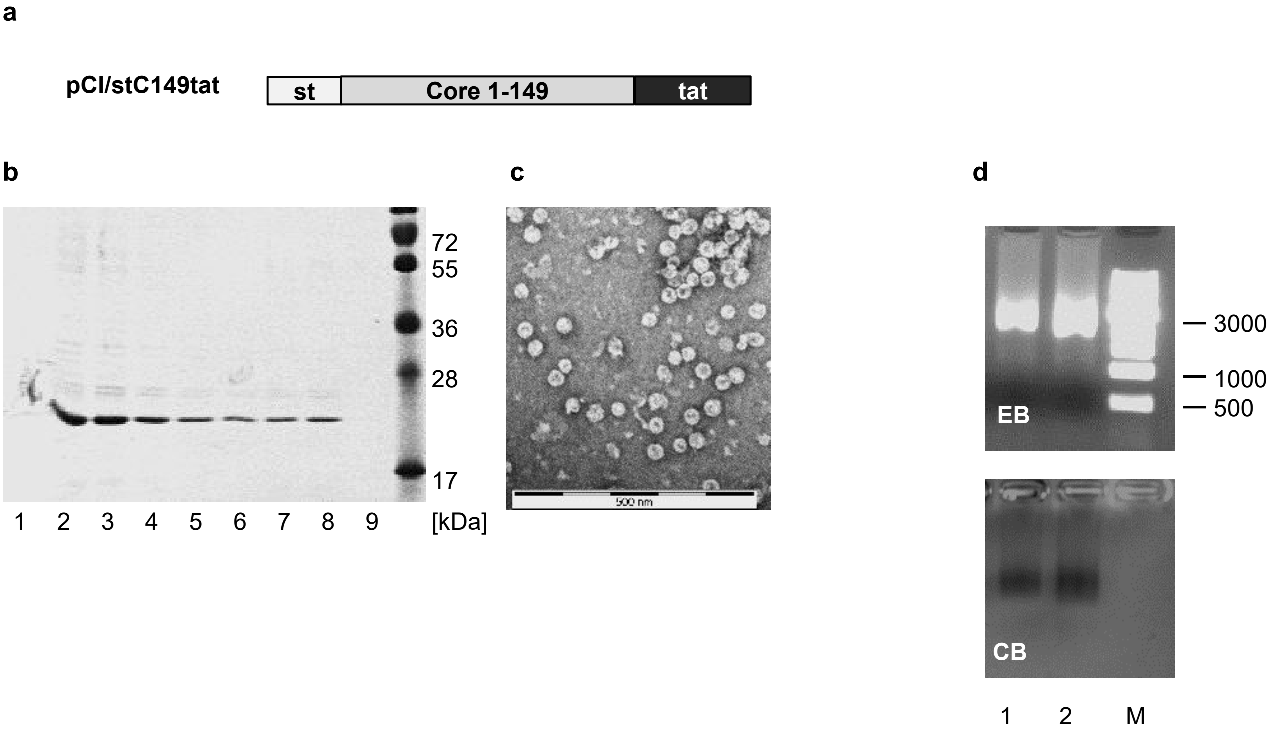

Supplementary Fig. S4

Characterization of HBV-stC149tat particles expressed in HEK-293 cells

(a) Schematic presentation of the HBV-stC149tat antigen. The strep-tag (st) and the HIV-tat domain are indicated. **(b)** HEK-293 cells were transiently transfected with pCI/stC149tat plasmid and recombinant protein was purified as described in M&M section. 10 µl of elution fractions (fractions 1 to 9) were processed for SDS-PAGE followed by Coomassie blue staining of the gel. The molecular weight marker is shown. (**c**) 2µg of purified protein was analysed on Joel TEM1400 at 100kV. The scale bar represents 500nm. (**d**) Two different HBV-stC149tat antigen preparations (1 and 2) were run on native agarose gels and stained with ethidium bromide (EB; upper panel) followed by Coomassie blue staining (CB; lower panel).

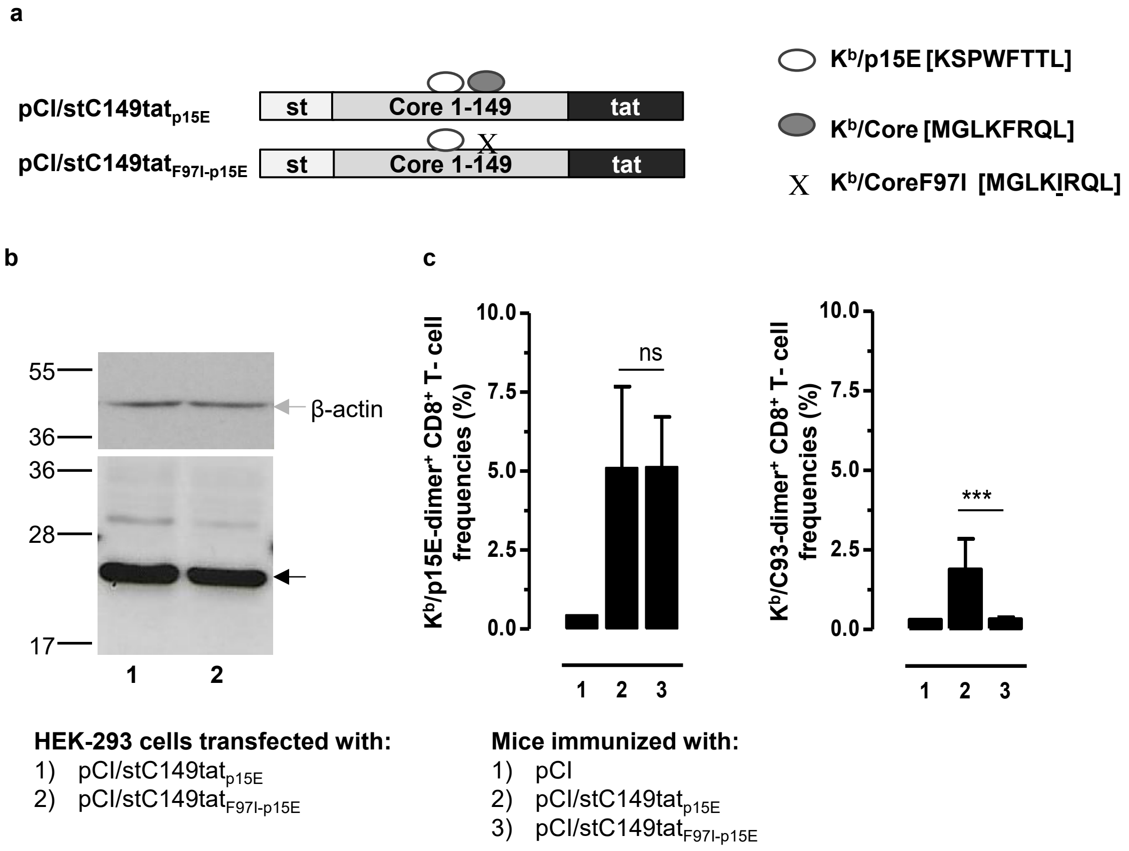

Supplementary Fig. S5

Induction of K^b/p15E-specific $CD8^+$ T-cell responses by DNA-based vaccines encoding chimeric HBV-C/p15E antigens

(a) Schematic presentation of pCI/stC149tat$_{p15E}$ and pCI/stC149tat$_{F97I-p15E}$ antigens. The chimeric pCI/stC149tat$_{F97I-p15E}$ antigen harboring the K^b/p15E and the silenced K^b/C93$_{F97I}$ epitopes is described in the result section and in legend to figure 2. Similarly, we constructed the pCI/stC149tat$_{p15E}$ vector encoding the chimeric HBV-stC149tat antigen with the K^b/p15E and the functional K^b/C93 epitopes. The K^b/p15E epitope, the K^b/C93 epitope and the mutant K^b/C93$_{F97I}$ amino acid sequence harboring the point mutation at position C97 (exchange of an F to an I) are indicated. **(b)** HEK-293 cells were transiently transfected with pCI/stC149tat$_{p15E}$ and pCI/stC149tat$_{F97I-p15E}$. Expression of the antigens was determined in cell lysates by western blot using strep-tag (black arrow) and beta-actin (gray arrow) specific antibodies. **(c)** B6 mice were immunized i.m. with pCI (group 1), pCI/stC149tat$_{p15E}$ (group 2; n=4) and pCI/stC149tat$_{F97I-p15E}$ (group 3; n=4). Twelve days post immunization, K^b/p15E-dimer$^+$ (left panel) and K^b/C93-dimer$^+$ (right panel) $CD8^+$ T-cell frequencies were determined in the spleen as described in the M&M section. The mean % of dimer$^+$ $CD8^+$ T cells in the splenic $CD8^+$ T-cell populations (+SD) are shown. The statistical significance of differences between groups 2 and 3 were determined by the unpaired Student's t-test. P values of $p < 0.001$ (***) were considered statistically significant. ns; not significant
