# Supplementary material for: Endogenously Expressed Antigens Bind Mammalian RNA via Cationic Domains that Enhance Priming of Effector CD8 T Cells by DNA Vaccination
Source: Mol Ther. 2019 Jan 22;27(3):661–72. doi: 10.1016/j.ymthe.2019.01.011 (PMC6403493; doi:10.1016/j.ymthe.2019.01.011)
Supplement: Document S2. Article plus Supplemental Information [file mmc2.pdf]

Molecular Therapy
Original Article

Endogenously Expressed Antigens Bind Mammalian RNA via Cationic Domains that Enhance Priming of Effector CD8 T Cells by DNA Vaccination

Jana Krieger,[1,3] Petra Riedl,[1,3] Katja Stifter,[1] Gleyder Roman-Sosa,[1,2] Thomas Seufferlein,[1] Martin Wagner,[1] and Reinhold Schirmbeck[1]

[1]Department of Internal Medicine I, Ulm University Hospital, Albert Einstein Allee 23, 89081 Ulm, Germany

Hepatitis B virus (HBV) core (HBV-C) antigens with homologous or heterologous HIV-tat48-57-like (HBV-C149tat) cationic domains non-specifically bind cellular RNA in vector-transfected cells. Here, we investigated whether RNA-binding to cationic domains influences the immunogenicity of endogenously expressed antigens delivered by DNA vaccination. We initially evaluated induction of HBV-C (K^b/C93)-specific $CD8^+$ T cell responses in C57BL/6J (B6) and 1.4HBV-S^{mut} transgenic (tg) mice that harbor a replicating HBV genome in hepatocytes by DNA immunization. RNA-binding HBV-C and HBV-C149tat antigens moderately enhanced K^b/C93-specific $CD8^+$ T cells in B6 mice as compared with RNA-free HBV-C149 antigen (lacking cationic domains). However, only the RNA-binding antigens elicited K^b/C93-specific $CD8^+$ T cells that inhibited HBV replication in 1.4HBV-S^{mut} tg mice. Moreover, RNA-binding to designer antigens, which express a K^b/p15E epitope from an endogenous murine leukemia virus-derived tumor-specific gp70 protein, was crucial to prime tumor-rejecting effector $CD8^+$ T cells in B6 mice. Antigen-bound endogenous RNAs function as a Toll-like receptor 7 (TLR-7) ligand and stimulated priming of K^b/p15E-specific $CD8^+$ T cells in B6, but not TLR-$7^{-/-}$, mice. Antigen-bound cellular RNAs thus function as an endogenous natural adjuvant in *in vivo* vector-transfected cells, and thus are an attractive tool to induce and/or enhance effector $CD8^+$ T cell responses directed against chronic viral infections or tumor self-antigens by DNA vaccination.

INTRODUCTION

It has been shown that non-specific "exogenous" bacterial RNAs function as a Toll-like receptor 7 (TLR-7) ligand and stimulated Th1-biased immune responses in mice, when co-delivered with recombinant antigens or when directly bound by particulate or non-particulate antigens.[1–6] Antigen-bound bacterial RNA has an >1,000-fold higher potency as a Th1-inducing adjuvant than free RNA mixed to a recombinant antigen.[7] Under certain conditions, mammalian self-RNAs also stimulated TLR-7- or TLR-3-mediated autoreactive B cell responses.[8–11] In particular, endogenous nucleic acids released from damaged cells can induce TLR-3- or TLR-7-mediated inflammation and stimulate and/or attract cells of the innate immune system.[11,12] Cellular RNAs thus could function as molecular adjuvant and stimulate cellular and humoral immune responses when targeted by vaccines.[13] In particular, DNA vaccines expressing RNA-binding antigen(s) are attractive to target immune-stimulating cellular RNA in *in vivo* transfected antigen-presenting cells.[14,15]

The 183-residue hepatitis B virus core (HBV-C) protein is an attractive model antigen to test immune-stimulatory functions of antigen-bound cellular RNA. When selectively expressed in bacterial, yeast, or mammalian expression systems, HBV-C protein self-assembled into particles that non-specifically bound heterologous RNAs.[1,2,4,6,16,17] The 34-residue COOH-terminal cationic domain of HBV-C (C150–183) is crucial for the non-specific RNA-binding of HBV-C particles, whereas HBV-C149 particles (lacking the cationic domain) did not bind RNA.[1,2,4,6,16,17] Non-phosphorylated HBV-C particles encapsidate high amounts of bacterial RNA but low amounts of mammalian RNA.[6,17] Prevention of specific phosphorylation in the cationic C150–183 domain by exchanging serine residues S155, S162, and S170 with alanine[6,17–19] or by exchanging the cationic C150–183 domain with a heterologous 14-residue HIV-tat$_{48–57}$-like cationic domain (HBV-C149tat), lacking any phosphorylation sites, significantly enhanced the RNA-binding of these mutant core particles.[6] Similarly, mammalian RNA efficiently bound to freely exposed cationic domains in assembly-deficient core antigens, indicating that stable RNA-binding primarily depends on interactions between positively charged cationic domains and negatively charged nucleic acids.[6,7] Both bacterial and mammalian RNAs bound to recombinant core particles (exogenous protein vaccines) or cellular RNAs bound to endogenously expressed core particles (endogenous DNA vaccines) function as TLR-7, but not TLR-3, ligands and induced a Th1-biased

Received 20 September 2018; accepted 11 January 2019;
https://doi.org/10.1016/j.ymthe.2019.01.011.

[2]Current Address: Unit of Structural Virology, Institut Pasteur, 25-28 Rue du Docteur Roux, 75015 Paris, France

[3]These authors contributed equally to this work.

Correspondence: Reinhold Schirmbeck, Department of Internal Medicine I, Ulm University Hospital, Albert Einstein Allee 23, 89081 Ulm, Germany.
E-mail: reinhold.schirmbeck@uni-ulm.de

This is an open access article under the CC BY license (http://creativecommons.org/licenses/by/4.0/).

humoral immunity in C57Bl6/J (B6) and TLR-3$^{-/-}$, but not in TLR-7$^{-/-}$, mice.[1,2,6]

Little is known whether mammalian RNAs also function as a natural adjuvant for priming effector CD8$^+$ T cell responses by DNA-based vaccines. Endogenously expressed HBV-C particles induced CD8$^+$ T cell responses that mediate HBV clearance in murine infection models.[20–22] Similarly, we could induce HBV-C-specific, but not HBV surface-specific, CD8$^+$ T cells in 1.4HBV-S^{mut} tg mice that harbor a replicating HBV genome in hepatocytes by DNA vaccination.[23–25] A single injection of the HBV-C expression vector pCI/C induced K^b/C93-specific CD8$^+$ T cells in 1.4HBV-S^{mut} tg mice. Dimer$^+$ K^b/C93-specific CD8$^+$ T cells accumulated in the liver but were barely detectable in the spleen of 1.4HBV-S^{mut} tg mice.[25] K^b/C93-specific CD8$^+$ T cells in 1.4HBV-S^{mut} tg mice, but not in B6 mice, largely lost production of interferon (IFN)-γ and upregulated cell surface expression of programmed cell death protein 1 (PD-1),[24,25] indicating that they gain an exhausted phenotype.[26] However, the K^b/C93-specific CD8$^+$ T cell response in 1.4HBV-S^{mut} tg mice was functional and, at least transiently, inhibited HBV replication in the liver.[24,25] We thus hypothesized that the binding of cellular RNA to endogenously expressed HBV-C plays a crucial role for priming of antiviral CD8$^+$ T cells in 1.4HBV-S^{mut} tg mice.

In this study, we tested *de novo* priming of antiviral K^b/C93-specific effector CD8$^+$ T cells in 1.4HBV-S^{mut} tg mice by DNA vaccines expressing RNA-binding or RNA-free HBV core antigens. We further analyzed whether expression of K^b/p15E or L^d/AH1 epitopes, from a tumor-specific envelope gp70 antigen of an endogenous murine leukemia virus (AKV),[27–31] in RNA-binding model antigens affects priming of effector CD8$^+$ T cells in mice by DNA vaccination.

RESULTS

HBV-C Antigens with an RNA-Capturing Cationic Domain Induce Antiviral K^b/C93-Specific CD8$^+$ T Cells in 1.4HBV-S^{mut} tg Mice by DNA Vaccination

We previously showed that HBV-C, but not the HBV-C149 antigen (lacking the cationic C150–183 domain), bound mammalian RNA in transiently transfected cell lines. To elucidate RNA-mediated helper function(s) on *de novo* priming of HBV-C (K^b/C93)-specific CD8$^+$ T cells, we initially immunized B6 mice with vectors that express the RNA-binding HBV-C (pCI/C) or the RNA-free HBV-C149 antigen (pCI/C149) (Figure 1A). Both vectors efficiently expressed HBV-C and HBV-C149 antigens in transiently transfected HEK293 cells (Figure 1B, lanes 1 and 3; Figure S1). A single injection of pCI/C into B6 mice tends to induce higher dimer$^+$ K^b/C93-specific CD8$^+$ T cell frequencies in the liver than the pCI/C149 vector (Figure 1C). Similarly, IFN-γ$^+$ K^b/C93-specific CD8$^+$ T cell frequencies, determined by *ex vivo* stimulation of spleen cells with the K^b/C93 peptide, were somewhat higher in pCI/C- than in pCI/C149-immune B6 mice (Figure 1C).

Next, we vaccinated 1.4HBV-S^{mut} tg mice with pCI/C and pCI/C149 vector DNAs. We confirmed that, at day 12 post-priming (i.e., the time point at which clonal expansion of *de novo* primed CD8$^+$ T cells reaches maximal levels), dimer$^+$ K^b/C93-specific CD8$^+$ T cells accumulated in the liver of pCI/C-immune 1.4HBV-S^{mut} tg mice and HBV replication was efficiently suppressed (Figures 1D and 1E, group 2).[24,25] In contrast, the pCI/C149 vector did not (or very inefficient) induce K^b/C93-specific CD8$^+$ T cells in 1.4HBV-S^{mut} tg mice, and HBV replication was not suppressed (Figures 1D and 1E, group 3). This showed that the helper function of cellular RNA bound to the cationic C150–183 domain of endogenously expressed HBV-C substantially enhanced priming and/or expansion of K^b/C93-specific CD8$^+$ T cells in 1.4HBV-S^{mut} tg mice.

To investigate whether the cationic C150–183 domain is crucial to induce antiviral CD8$^+$ T cells in 1.4HBV-S^{mut} tg mice, we used a pCI/C149tat vector composed of the HBV-C149 antigen and a short COOH-terminal cationic HIV-tat$_{48-57}$-like sequence (GRKKRRQRRRRRQ; https://www.uniprot.org/uniprot/P04610) (Figure 1A). This antigen self-assembled into particles and efficiently bound mammalian RNAs in transiently transfected HEK293 cells.[6] Comparable amounts of HBV-C149tat and HBV-C proteins were expressed in transiently transfected HEK293 cells (Figure 1B, lanes 1 and 2; Figure S1). Both pCI/C and pCI/C149tat vaccines induced comparable K^b/C93-specific CD8$^+$ T cell frequencies in the liver of 1.4HBV-S^{mut} tg mice and efficiently suppressed HBV replication (Figures 1D and 1E, groups 2 and 4). Thus, RNA bound to different cationic domains facilitated priming of antiviral K^b/C93-specific effector CD8$^+$ T cells in 1.4HBV-S^{mut} tg mice.

To confirm that the RNA-mediated "helper" function directly stimulates K^b/C93-monospecific CD8$^+$ T cells that suppress HBV replication in 1.4HBV-S^{mut} tg mice, we silenced the K^b/C93 epitope in the pCI/C vector by exchanging the phenylalanine (F) with an isoleucine (I) residue in the central K^b-binding anchor motif (MGLK$^{\underline{F}}$**I**RQL).[32] The newly generated pCI/C$_{F97I}$ and the pCI/C vector expressed comparable amounts of antigens in transiently transfected HEK293 cells (Figures S2A and S2B). However, the pCI/C$_{F97I}$ vaccine did neither induce CD8$^+$ T cells in 1.4HBV-S^{mut} tg and B6 mice (Figure S2C) nor inhibit HBV replication in 1.4HBV-S^{mut} tg mice (Figure S2D). Similarly, a pCI/Cadw2 vector expressing a natural HBV-C variant that contains this C$_{F97I}$ mutation and additional five amino acid mismatches did not induce antiviral K^b/C93-specific CD8$^+$ T cells in B6 and 1.4HBV-S^{mut} tg mice.[24] K^b/C93-monospecific effector CD8$^+$ T cells thus are the main players in the pCI/C-induced inhibition of HBV replication in 1.4HBV-S^{mut} tg mice.

Priming of gp70 K^b/p15E-Specific CD8$^+$ T Cells in B6 Mice by Vectors Expressing Chimeric RNA-Binding Core Antigens

To evaluate whether binding of cellular RNA to endogenously expressed antigens could enhance *de novo* priming of CD8$^+$ T cells in B6 mice, we chose a tumor-specific envelope glycoprotein (gp70) of an endogenous murine leukemia virus (AKV) that integrated as a provirus in the mouse germline DNA (https://www.uniprot.org/uniprot/P03386; https://www.genome.jp/dbget-bin/www_bget?genbank-vrl:J01998). This protein contains two well-defined K^b/p15E

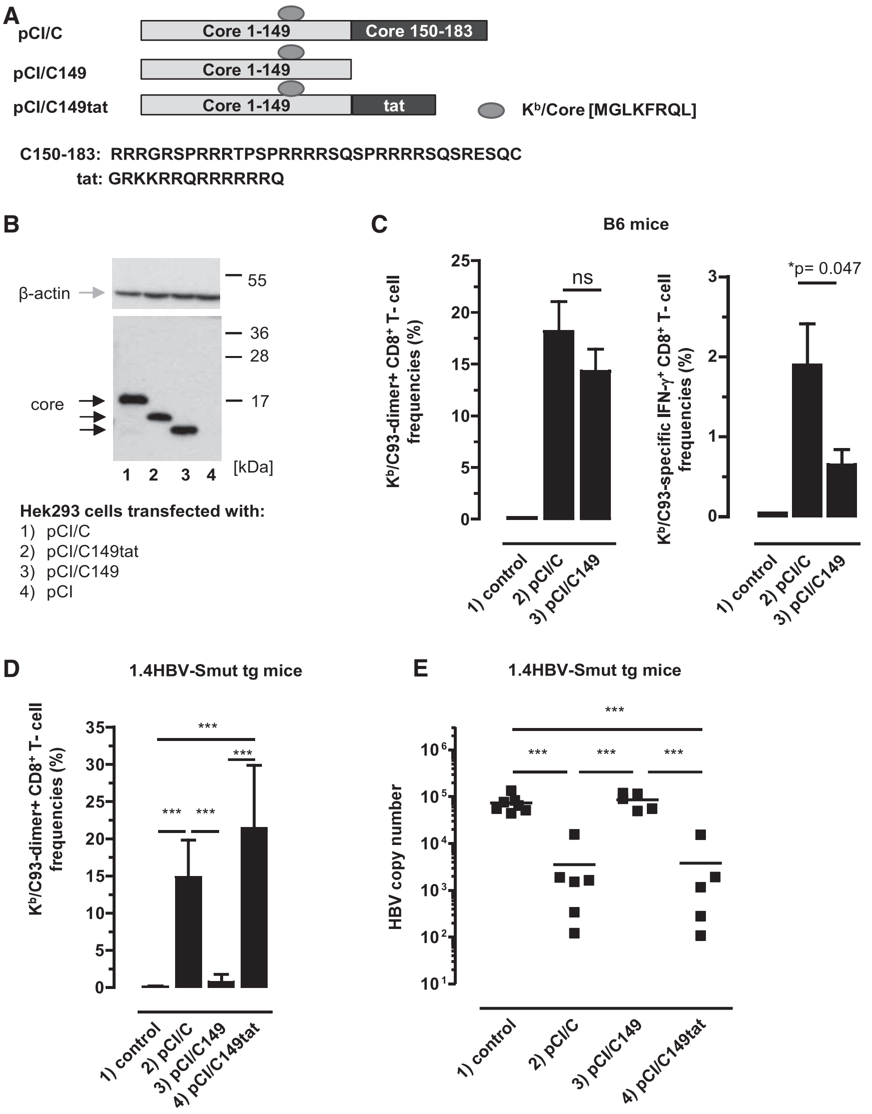

Figure 1. Induction of K^b/C93-Specific $CD8^+$ T Cell Responses by DNA-Based Vaccines

(A) Schematic presentation of HBV-C, HBV-C149, and HBV-C149tat antigens. The position and amino acid (aa) sequences of the K^b/C93-100 epitope, the C150–183, and HIV-tat-derived cationic domains are given. (B) HEK293 cells were transiently transfected with the indicated plasmids, lysed, and processed for SDS-PAGE followed by core- and actin-specific western blot analyses. The positions of β-actin (gray arrow), HBV core antigens (black arrows), and a molecular weight marker (in kDa) are indicated. This image was generated from two films with different exposure times (1 min for the lower part and 3 min for the upper part). (C) B6 mice were either non-treated (group 1; n = 4) or injected with pCI/C (group 2; n = 6) and pCI/C149 (group 3; n = 6). Twelve days post-immunization, K^b/C93-specific dimer$^+$ $CD8^+$ T cell responses were determined in the liver. The mean percentage of K^b/C93-specific dimer$^+$ $CD8^+$ T cells in the hepatic $CD8^+$ T cell populations (±SD) is shown (left panel). Furthermore, spleen cells were *ex vivo* re-stimulated with K^b/C93 or an irrelevant K^b/Ova257-264 peptide. Specific IFN-γ^+ $CD8^+$ T cell frequencies were determined by FCM, and K^b/Ova257-specific background values were subtracted. The mean percentage of K^b/C93-specific IFN-γ^+ $CD8^+$ T cells in the splenic $CD8^+$ T cell population (±SD) is shown (right panel). The statistical significance of differences in dimer$^+$ or IFN-γ^+ $CD8^+$ T cell frequencies between groups 2 and 3 was determined by the unpaired Student's t test. *p values <0.05 were considered statistically significant. (D and E) 1.4HBV-S^{mut} tg mice (four to six mice per group) were either non-treated (group 1) or injected with pCI/C (group 2), pCI/C149 (group 3), and pCI/C149tat (group 4) DNA. (D) Twelve days post-immunization, K^b/C93-specific dimer$^+$ $CD8^+$ T cell responses were determined in the liver. The mean percentage of K^b/C93-specific dimer$^+$ $CD8^+$ T cells in the hepatic $CD8^+$ T cell populations (±SD) is shown. (E) HBV replication was determined in the liver of non-treated (control) and vaccinated 1.4HBV-S^{mut} tg mice by real-time qPCR as described in the Materials and Methods. (D and E) The statistical significance of differences between different groups of 1.4HBV-S^{mut} tg mice was determined by one-way ANOVA followed by Tukey's multiple comparison test. *$p < 0.05$ and ***$p < 0.001$ were considered significant. ns, not significant.

(KSPWFTTL) and L^d/AH1 (SPSYVYHQF) epitopes and was expected to present a low immunogenicity.[33–35] We generated a pCI/stgp70 vector, composed of an NH_2-terminal streptavidin-binding tag (st or strep) and a 289-residue $gp70_{327–615}$ fragment (Figure 2A). The stgp70 protein was efficiently expressed in transiently pCI/stgp70 vector-transfected HEK293 cells (Figure 2B, lane 2), but the pCI/stgp70 vector inefficiently induced K^b/p15E-specific $CD8^+$ T cells in B6 mice. K^b/p15E-specific dimer$^+$ $CD8^+$ T cells were unequivocally detectable in the spleen of two out of six pCI/stgp70-immune mice B6 mice (Figure 2C; Figure S3), and IFN-γ^+ K^b/p15E-specific $CD8^+$ T cells were not detectable (Figure 2C). This confirmed the very low immunogenicity of gp70 antigens delivered by DNA vaccines.[33–35]

We next asked whether the RNA-binding HBV-stC149tat antigen could be used as carrier for the K^b/p15E epitope to enhance K^b/p15E-specific $CD8^+$ T cell responses in B6 mice. To exclude that co-priming of K^b/C93-specific $CD8^+$ T cells affects the K^b/p15E-specific T cell response, we cloned the sequence of the K^b/p15E epitope into the major immunodominant region (MIR) between C78 and C81[36] of an HBV-stC149tat$_{F97I}$ antigen encoding the silenced K^b/C93$_{F97I}$ epitope (pCI/stC149tat$_{F97I-p15E}$) (Figure 2A). Using st-specific western blotting, we showed that comparable steady-state levels of HBV-stC149tat$_{F97I-p15E}$ and stgp70 proteins were expressed in HEK293 cells transiently transfected with the respective vectors (Figure 2B). The frequencies of dimer$^+$ and IFN-γ^+ K^b/p15E-specific $CD8^+$ T cells were significantly enhanced

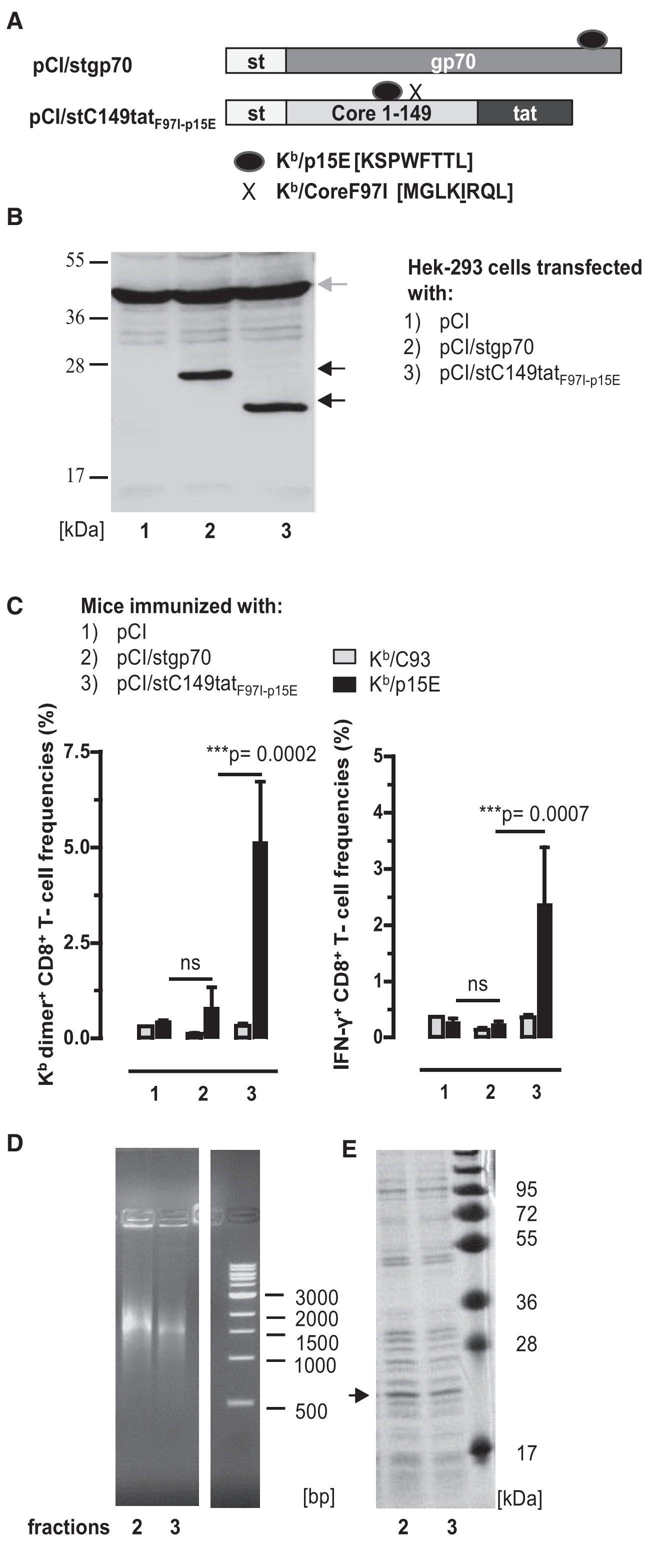

Figure 2. Induction of K^b/p15E-Specific CD8$^+$ T Cell Responses by DNA Vaccination

(A) Schematic presentation of pCI/stgp70 and pCI/stC149tat$_{F97I\text{-}p15E}$ antigens. The pCI/stgp70 vector is composed of an NH$_2$-terminal strep-tag (st) and a 289-residue gp70$_{327–615}$ fragment. PCI/stC149tat$_{F97I\text{-}p15E}$ encodes for the HBV-stC149tat antigen with the K^b/p15E sequence cloned into the major immunodominant region. Additionally, it harbors a point mutation at position 97 in the HBV-C encoding region, silencing the K^b/C93 epitope. The corresponding K^b/C93$_{F97I}$ sequence is indicated by an X. (B) Lysates of HEK293 cells transiently transfected with pCI/stgp70 and pCI/stC149tat$_{F97I\text{-}p15E}$ were processed for SDS-PAGE followed by strep-tag- (black arrows) and actin-specific (gray arrow) western blot analyses. The position of the molecular weight marker (in kDa) is indicated. (C) B6 mice were immunized intramuscularly (i.m.) with pCI (group 1; n = 3), pCI/stgp70 (group 2; n = 6), and pCI/stC149tat$_{F97I\text{-}p15E}$ (group 3; n = 4). Twelve days post-immunization, antigen-specific (K^b/p15E and K^b/C93) dimer$^+$ and IFN-γ^+ CD8$^+$ T cell frequencies were determined in the spleen by FCM. The mean percentages of dimer$^+$ and IFN-γ^+ CD8$^+$ T cells in the splenic CD8$^+$ T cell populations (+SD) are shown. The statistical significance of differences in K^b/p15E-specific frequencies between indicated groups was determined by the unpaired Student's t test. *p < 0.05, **p < 0.01, and ***p < 0.001 were considered statistically significant. (D and E) HEK293 cells were transiently transfected with the pCI/stC149tat$_{F97I\text{-}p15E}$. Recombinant stCtat$_{F97I\text{-}p15E}$ antigen was purified from cell lysates, and elution fractions 2 and 3 were analyzed on a native agarose gel stained with ethidium bromide (EB) (D) and on an SDS-containing polyacrylamide gel stained with coomassie blue (CB) (E). The molecular weight marker (in kDa) and DNA marker (in bp) are shown. ns, not significant.

in pCI/stC149tat$_{F97I\text{-}p15E}$-immune B6 mice as compared with pCI/stgp70-immune B6 mice (Figure 2C, groups 2 and 3). As expected, K^b/C93-specific CD8$^+$ T cells were not detectable in pCI/stC149tat$_{F97I\text{-}p15E}$-immune B6 mice (Figure 2C).

To confirm that *de novo* priming of K^b/p15E-specific CD8$^+$ T cells depends on the RNA-binding of the chimeric HBV-stC149tat$_{F97I\text{-}p15E}$ antigen, we expressed this protein in transiently transfected HEK293 cells. For large-scale production, we transiently transfected 8×10^8 HEK293 cells with the pCI/stC149tat$_{F97I\text{-}p15E}$ vector, and recombinant HBV-stC149tat$_{F97I\text{-}p15E}$ antigen was isolated from lysates by st-specific protein purification. Purified HBV-stC149tat$_{F97I\text{-}p15E}$ samples efficiently bound mammalian RNA (Figure 2D), but also contained a large number of co-purifying cellular proteins (Figure 2E). HBV-stC149tat$_{F97I\text{-}p15E}$ particles were not detectable by electron microscopy (data not shown), indicating that this protein is assembly deficient. In contrast, we confirmed that the HBV-stC149tat antigen self-assembled into particles that exclusively encapsidated cellular RNA (Figure S4).[6] Efficient binding of both cellular RNA and RNA-binding proteins is typically seen in samples of non-particulate core antigens, in which the cationic domain(s) is freely exposed in the cytosol and nucleus of transfected cells.[6] RNase A treatment of such protein preparations quantitatively destroyed the bound RNA and removed most of the co-precipitated cellular proteins. This showed that cellular proteins bind to RNA initially captured by antigen-specific cationic domains, but the subsequent binding of RNA-binding proteins may proceed non-specifically during protein purification in the cell lysates.[6]

Overall, this showed that the RNA-binding non-particulate HBV-stC149tat$_{F97I\text{-}p15E}$ antigen can be used as carrier for the K^b/p15E

epitope to efficiently prime K^b/p15E-specific $CD8^+$ T cell responses in B6 mice by DNA vaccination. Similarly, a pCI/stC149tat$_{p15E}$ vaccine, differing from pCI/stC149tat$_{F97I-p15E}$ in the reversion of the mutant K^b/C93$_{F97I}$ back to the wild-type K^b/C93 epitope, primed K^b/p15E-specific $CD8^+$ T cells in vaccinated B6 mice, irrespective of whether K^b/C93-specific $CD8^+$ T cells were co-primed or not (Figures S5A–S5C).

Fusion of the RNA-Binding Cationic tat Domain to the stgp70 Antigen Is Sufficient to Enhance Priming of K^b/p15E-Specific $CD8^+$ T Cells in B6 Mice

The above findings indicated that RNA-binding to non-particulate HBV-stC149tat$_{F97I-p15E}$ or HBV-stC149tat$_{p15E}$ antigens was crucial for efficient priming of K^b/p15E-specific $CD8^+$ T cells by DNA vaccination (Figure 2; Figure S5). To show directly that the RNA-binding cationic tat domain has an impact on *de novo* priming of K^b/p15E-specific $CD8^+$ T cells, we next fused this domain COOH-terminally to the stgp70 antigen, generating the pCI/stgp70tat vector (Figure 3A). Both pCI/stgp70 and pCI/stgp70tat vectors expressed comparable steady-state levels of the respective antigens in transiently transfected HEK293 cells, as determined in total cell lysates of 5×10^5 transfected cells by st-specific western blotting (Figure 3B). To determine the RNA-binding capacity of these proteins, we produced recombinant stgp70tat and stgp70 proteins from lysates of the same numbers of transiently transfected HEK293 cells by st-specific protein purification. We determined a somewhat higher level of the stgp70 protein as compared with the stgp70tat protein (Figures 3C and 3D). However, mammalian RNA bound to stgp70tat, but not to stgp70 protein (Figure 3D). The pCI/stgp70tat vaccine induced significantly higher frequencies of K^b/p15E-specific $CD8^+$ T cells in B6 mice, as well as L^d/AH1-specific $CD8^+$ T cells in H-2^d BALB/c mice, than the pCI/stgp70 vector (Figures 3E and 3F). The efficient priming of gp70-specific $CD8^+$ T cells by pCI/stgp70tat thus exclusively depends on cellular RNA bound to the cationic tat domain.

The pCI/stgp70tat Vaccine Induced gp70 Tumor-Specific Effector $CD8^+$ T Cells

To further confirm that K^b/p15E-specific $CD8^+$ T cells are functional in vaccinated mice, we analyzed their *in vivo* cytotoxicity in vaccinated B6 mice. B6 mice were injected with pCI/stgp70tat or control pCI vectors. Spleen cells of non-treated B6 mice were pulsed with K^b/p15E (carboxyfluorescein succinimidyl ester $[CFSE]^{hi}$) and irrelevant K^b/Ova257 ($CFSE^{low}$) peptides, mixed at a 1:1 ratio, and injected into B6 mice at day 12 post-immunization. The data shown in Figure 4 clearly demonstrated that priming of $dimer^+$ K^b/p15E-specific $CD8^+$ T cells in pCI/stgp70tat-immune B6 mice correlated with an almost quantitative elimination of K^b/p15E-presenting spleen cells.

Using well-defined primer pairs, we could not detect expression of AKV-gp70 mRNA in the thymus or spleen of BALB/c and B6 mice (Figures 5A and 5B).[29,31,37] However, retroviral gp70 mRNA was efficiently expressed in human and murine tumors and tumor cell lines,[38] for example, in the BALB/c-derived colon carcinoma CT26[29] and in B6-derived pancreatic ductal adenocarcinoma (AKC-5615 cells), established from a highly aggressive tumor in ATM-deficient AKC mice ($Atm^{lox/lox}$;$Kras^{LSL-G12D/+}$;p48Cre; $Atm^{lox/lox}$ (Figures 5A and 5B).[39] To confirm that the expression of the RNA-binding tat domain in the stgp70tat antigen is sufficient to induce tumor-specific effector $CD8^+$ T cell responses, we vaccinated B6 mice with pCI, pCI/stgp70, or pCI/stgp70tat DNA. At day 12 post-injection, mice were challenged subcutaneously with 5×10^5 gp70-expressing AKC-5615 cells (Figure 5C). Transplantation of 5×10^5 AKC-5615 cells gave rise to tumors in transplanted pCI- and pCI/stgp70-immune animals (Figure 5C). In contrast, vaccination of mice with pCI/stgp70tat efficiently inhibited outgrowth of tumors (Figure 5C). Similarly, the pCI/stgp70tat, but not pCI and pCI/stgp70, vaccines efficiently suppressed outgrowth of 1×10^6 CT26 cells in BALB/c mice (Figure 5D). Elimination of tumor transplants thus correlated with the priming of K^b/p15E- and L^d/AH1-specific $CD8^+$ T cells by pCI/stgp70tat DNA (Figures 3E and 3F). This showed that a tumor-specific effector $CD8^+$ T cell immunity to gp70 (K^b/p15E and L^d/AH1) epitopes was primed in different mouse strains by the endogenously expressed RNA-binding stgp70tat, but not RNA-free stgp70 antigens.

Antigen-Bound Cellular RNAs Function as TLR-7 Ligand and Stimulate Priming of K^b/p15E-Specific $CD8^+$ T Cells in B6 Mice by DNA Vaccination

The above findings showed that *de novo* priming and/or expansion of gp70 (K^b/p15E)-specific $CD8^+$ T cells, but not HBV-C antigen (K^b/C93)-specific $CD8^+$ T cells, was substantially enhanced in B6 mice by DNA vaccines expressing RNA-binding antigens. Most interestingly, pCI/stC149tat$_{F97I-p15E}$ and pCI/stgp70tat vectors elicited K^b/p15E-specific $CD8^+$ T cells in B6, but not in TLR-$7^{-/-}$, mice (Figures 6A–6D). This clearly showed that antigen-bound endogenous RNAs function as TLR-7 ligand, and TLR-7-mediated helper responses were crucial for priming K^b/p15E-specific $CD8^+$ T cells. In contrast, a pCI/stC149tat vector elicited comparable K^b/C93-specific $CD8^+$ T cell frequencies in B6 and TLR-$7^{-/-}$ mice (Figures 6E and 6F). This suggested that, at least in B6 mice, a TLR-7-mediated helper function of antigen-bound endogenous RNA was not (or less) important for the induction of K^b/C93-specific $CD8^+$ T cells.

DISCUSSION

DNA vaccination is an attractive technique to elicit antigen-specific $CD8^+$ T cell responses in the host, because vector-encoded antigen expression and MHC class I-restricted epitope presentation directly proceed in *in vivo* transfected APCs.[14,15] Here, we describe a novel strategy to enhance priming of murine $CD8^+$ T cell responses by DNA vaccination. Vector-expressed particle-forming and non-particulate antigens containing homologous or heterologous cationic domains non-specifically capture mammalian RNA in transfected cells. *In vivo*, this antigen-bound cellular RNA functions as a natural endogenous adjuvant[12,13] and enhanced *de novo* priming of effector $CD8^+$ T cell responses in two well-defined mouse models by DNA vaccination: (1) K^b/C93-specific $CD8^+$ T cells directed against a transgenic HBV-C antigen in the liver of 1.4HBV-S^{mut} tg mice that suppressed HBV replication, and (2) K^b/p15E-specific $CD8^+$ T cells directed against a tumor-specific gp70 antigen that suppressed an

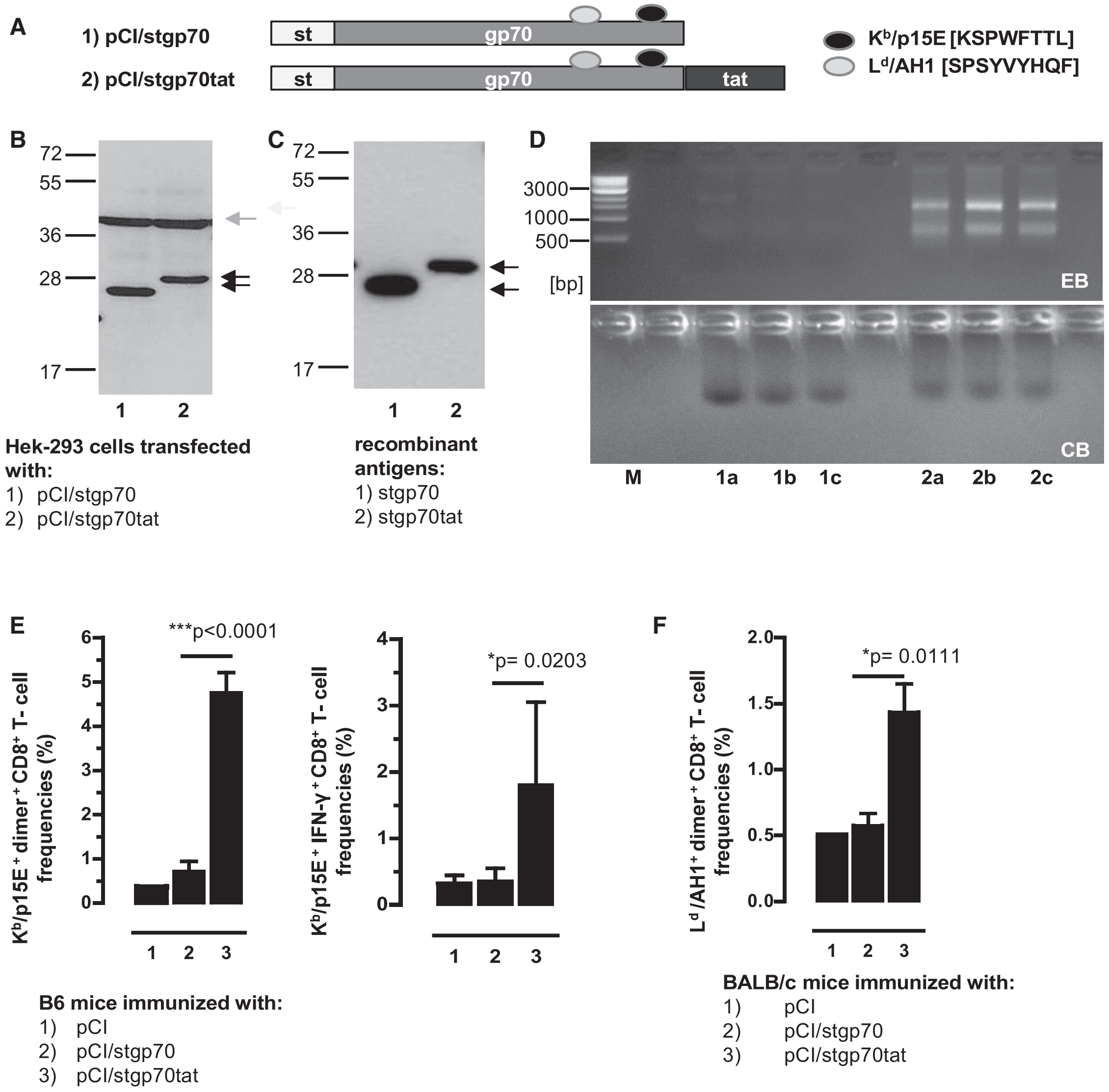

Figure 3. RNA-Binding of the stgp70tat Fusion Protein Facilitated Priming of gp70-Specific $CD8^+$ T Cell Responses

(A) Schematic presentation of stgp70 and stgp70tat antigens. The positions of the strep-tag (st), the $gp70_{327–615}$ fragment, and the HIV-tat-like cationic domain, as well as the L^d and K^b binding epitopes, are indicated. (B) Same numbers of HEK293 cells were transiently transfected with the pCI/stgp70 and pCI/stgp70tat vectors. Forty-eight hours post-transfection, cell lysates were processed for SDS-PAGE followed by strep-tag and β-actin-specific western blot. The position of the antigens (black arrows), β-actin (gray arrow), and the molecular weight marker (in kDa) are indicated. (C) Recombinant proteins were purified from lysates of the same numbers of transfected HEK293 cells as described in the Materials and Methods. Samples of pooled elution fractions (see D, lanes 1a, 1b, 1c and 2a, 2b, and 2c) were processed for SDS-PAGE, and antigens were detected by strep-tag-specific western blot. (D) Elution fractions of purified stgp70 (lanes 1a, 1b, and 1c) and stgp70tat (lanes 2a, 2b, and 2c) were analyzed by native agarose gel electrophoresis followed by EB (upper panel) and subsequent CB staining of the gel (lower panel). (E and F) B6 mice (five to seven mice per group) (E) and BALB/c mice (four mice per group) (F) were vaccinated with pCI (group 1), pCI/stgp70 (group 2), and pCI/stgp70tat (group 3) vectors. K^b/p15E-specific $dimer^+$ and IFN-γ^+ $CD8^+$ T cells (E) and L^d/AH1-specific $dimer^+$ $CD8^+$ T cells (F) in the spleen were determined 12 days post-injection by FCM. (E and F) The mean percentages of $dimer^+$ or IFN-γ^+ $CD8^+$ T cells in the splenic $CD8^+$ T cell populations (+SD) are shown. The statistical significance of differences between groups 2 and 3 were determined by the unpaired Student's t test. *$p < 0.05$ and ***$p < 0.001$ were considered statistically significant.

outgrowth of subcutaneously transplanted gp70-expressing tumor cells in B6 mice.

Retroviral mRNAs and antigens were expressed in human and murine tumors and tumor cell lines.[38] Attempts have been made to use the AKV gp70 tumor-specific antigen as a model for $CD8^+$ T cell-inducing cancer vaccines.[33–35] We previously developed an expression system in which chimeric proteins with a NH_2-terminal stress protein-capturing viral DnaJ-like sequence (J domain) fused to diverse antigen-encoding sequences to form stable complexes

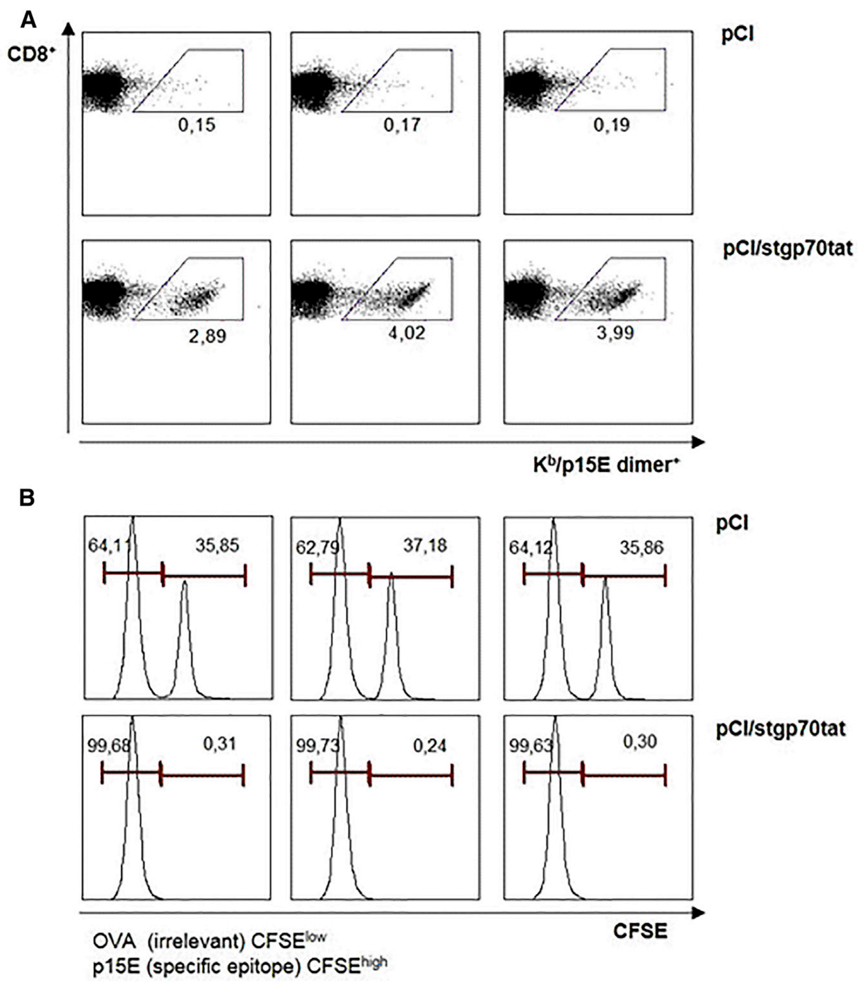

Figure 4. Antigen-Specific *In Vivo* Killing of Peptide-Pulsed Target Cells

Groups of B6 mice (n = 3) were vaccinated with pCI or pCI/stgp70tat vectors. Twelve days post-injection, peptide-pulsed and CFSE-labeled (irrelevant K^b/Oav257 peptide/low CFSE and K^b/p15E peptide/high CFSE) naive splenocytes of non-treated B6 mice were mixed and adoptively transferred (i.v.) into vaccinated recipients. Spleens were harvested 16 h post-transfer and analyzed for antigen-specific p15E dimer$^+$ CD8$^+$ T cells (A), and CSFE-labeled cells were quantified by FCM (B).

with constitutively expressed eukaryotic Hsp73 and accumulate to high steady-state levels in vector-transfected eukaryotic cells.[40,41] Hsp73-capturing, chimeric antigens displayed enhanced immunogenicity for T and B cells,[40] facilitated cross-priming of CD8$^+$ T cells to tumor cell antigens by dendritic cells (DCs),[42] and allowed priming of a gp70 (L^d/AH1)-specific CD8$^+$ T cell-mediated anti-tumor immunity in BALB/c mice by DNA vaccination.[35] Targeting of host-specific helper molecules like Hsp73 or cellular RNA (this study) by vector-expressed endogenous antigens is thus an attractive strategy to enhance *de novo* priming of effector CD8$^+$ T cell responses by DNA vaccination.

The mechanism(s) of RNA-mediated helper functions, relevant for CD8$^+$ T cell priming by DNA vaccination, are not yet fully understood. DNA vaccines efficiently prime CD8$^+$ T cells, because they allow expression of antigens in *in vivo* transduced APCs. However, it is largely unknown whether CD8$^+$ T cells were activated directly by professional APCs (e.g., DCs) targeted by intramuscular vector DNA injection and/or indirectly by antigens or antigenic material released from dying vector-transfected, antigen-expressing non-professional APCs (e.g., myocytes) and "cross-presented" to DCs.[43] We previously showed that RNA-bound, but not RNA-free, HBV-C antigens stimulated a Th1-biased, core-specific humoral immunity by DNA vaccination with the gene gun.[1,6] Considering that RNA-bound HBV-C particles were not secreted into the cell culture supernatant of transfected cells and that B cells require uptake of exogenous antigens for their activation, at least a small amount of RNA-bound core antigen must be released from *in vivo* transfected antigen-expressing cells to induce core-specific antibody responses. In this cross-presentation pathway, exogenous antigen-bound cellular RNA could stimulate the local induction of an inflammatory milieu, the activation of different arms of the innate immune system, and/or the attraction of professional APCs.[44] This could favor *de novo* priming of Th1-biased humoral immune responses by DNA vaccination, but its effects for priming CD8$^+$ T cells are not yet known. We could not induce K^b/C93-specific CD8$^+$ T cells in B6 or 1.4HBV-S^{mut} tg mice by exogenous recombinant RNA-bound HBV-C particles.[7] This suggested that endogenously expressed, but not recombinant, HBV-C antigens were efficiently ingested by DCs for efficient MHC class I presentation and activation of CD8$^+$ T cell responses.[2] This illustrated a major advantage of DNA vaccination for the induction of HBV-C-specific CD8$^+$ T cell responses.

Several virus-specific RNA motifs and polyuridylic (polyU) sequences that engage the TLR-7 receptor have been identified.[3,5,12] HBV-stC149-tat particles contained mammalian RNAs that varied in length from about 50 to 4,000 nt with no specific prevalence for small or large RNAs.[6] Yet it is unknown whether a specific RNA species and/or specific motifs within these RNA molecules engage the TLR-7. In future analyses, we will determine the specificity of antigen-bound cellular RNAs purified from transfected cells by next-generation sequencing (NGS). In comparison with the endogenous transcriptome of transfected cells, we expect to get hints if (and which) RNAs were specifically

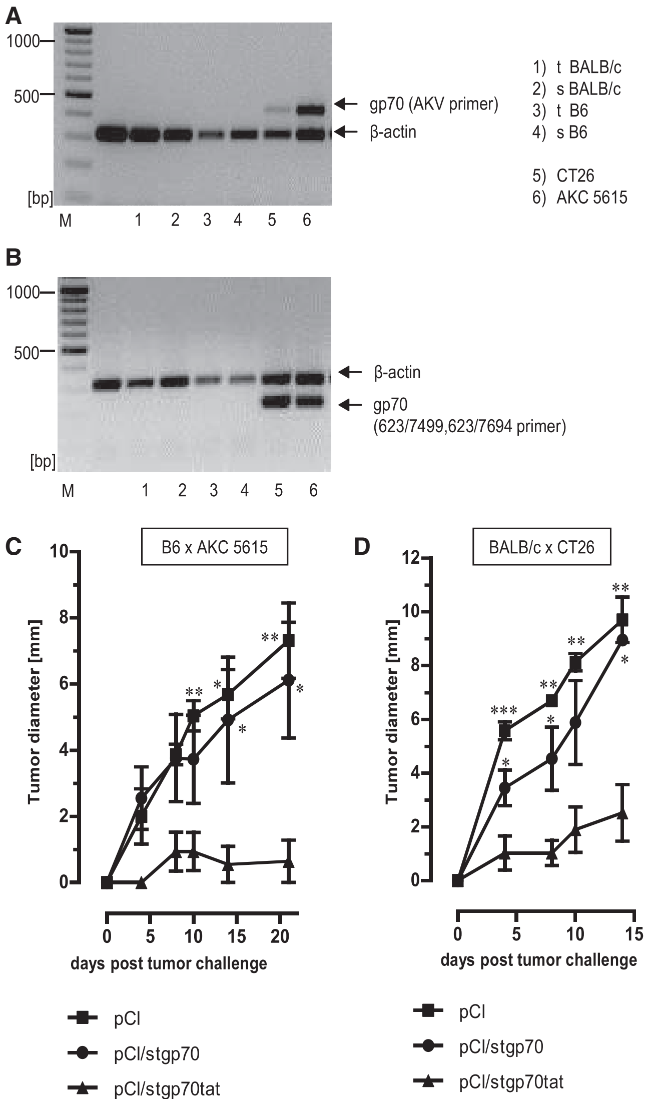

Figure 5. Rejection of gp70-Expressing CT26 and AKC-5615 Tumors in Vaccinated B6 and BALB/c Mice

(A and B) RNA was purified from thymus (t) and spleen (s) of B6 and BALB/c mice or CT26 and AKC-5615 cancer cell lines and transcribed into cDNA followed by PCR with gp70-specific AKV primers (A), 623/7499, 623/7694 primers (B), as well as β-actin-specific primers as described in the Materials and Methods. PCR products were analyzed on 1.5% agarose gels stained with EB. Positions of β-actin- and gp70-specific PCR products are indicated. (C) Growth of AKC-5615 tumors in B6 mice vaccinated with pCI/stgp70, pCI/stgp70tat, or pCI. Five mice per group were vaccinated with the indicated vectors, and 5 × 10^5 AKC-5615 cells were transplanted subcutaneously (s.c.) at 12 days post-vaccination. (D) Growth of CT26 tumors in BALB/c mice vaccinated with pCI/stgp70, pCI/stgp70tat, or pCI. Five mice per group were vaccinated and boosted (3 weeks after the first injection) with the indicated vectors. Mice were transplanted s.c. with 10^6 CT26 cells at day 12 after the second DNA injection. (C and D) Tumor growth was followed daily by serial measurements of tumor size at two perpendicular diameters. Mean values of five mice per group ± SEM are shown. The statistical significance of differences in tumor size between groups at the indicated time points was determined by one-way ANOVA followed by Tukey's multiple comparison test. *p < 0.05, **p < 0.01, and ***p < 0.001 were considered statistically significant.

targeted by different cationic domains present either in particulate or non-particulate antigens.

Here, we showed that antigen-bound endogenous RNAs function as TLR-7 ligand, and TLR-7-mediated helper function(s) was crucial for priming K^b/p15E-specific, but not K^b/C93-specific, $CD8^+$ T cells in B6 mice by DNA vaccination. Comparable frequencies of K^b/C93-specific $CD8^+$ T cells were primed in B6 and TLR-$7^{-/-}$ mice by pCI/stC149tat. In contrast, the pCI/stC149tat vector preferentially induced Th1-biased core-specific immunoglobulin G2 (IgG2) serum antibodies in B6 mice, but a balanced core-specific IgG1/IgG2b antibody profile in TLR-$7^{-/-}$ mice.[6] This showed that cellular RNAs captured by endogenously expressed HBV-stC149tat particles also function as a TLR-7 ligand and stimulated a Th1-biased humoral immunity in B6 mice,[6] but its TLR-7-mediated helper function was apparently not (or less) important for the induction of K^b/C93-specific $CD8^+$ T cells in B6 mice. We think that in addition to immune-stimulatory mechanisms by cellular RNA bound to endogenously expressed HBV-stC149tat particles, other signals, for example, from particle structures, could also function as PRRs[22,45,46] and may mask the specific effects of the RNA adjuvant in B6 mice. In contrast, endogenous RNA bound to HBV-C particles was crucial to induce effector $CD8^+$ T cells in 1.4HBV-S^{mut} tg mice that constitutively express the endogenous HBV-C antigen in the liver.[23] Antiviral K^b/C93-specific $CD8^+$ T cells are therefore induced under stringent conditions (i.e., operating against the tolerogenic milieu of an antigen-expressing liver)[47,48] and against potential self-antigen-specific tolerance mechanisms to the tg core protein[49] by DNA immunization. It is difficult to assign specific effects of the RNA-mediated adjuvant activity to K^b/C93-specific $CD8^+$ T cell responses in 1.4HBV-S^{mut} tg mice because these T cells circulate in the host, recognize their target antigen in the liver, and specifically respond to it. K^b/C93-specific $CD8^+$ T cells accumulated in the liver of 1.4HBV-S^{mut} tg mice and showed an exhausted phenotype.[26] Hence, RNA-mediated innate immune responses could also operate at the level of peripheral effector T cell homeostasis and/or delay the exhaustion of effector $CD8^+$ T cells in 1.4HBV-S^{mut} tg mice.

In summary, targeting an endogenous RNA adjuvant in APCs of a vaccine recipient by designer antigens expressing well-defined cationic domains may help to design new generations of DNA vaccines that efficiently prime $CD8^+$ T cell responses against chronic virus infections or tumors.

MATERIALS AND METHODS

Mice

All mouse immunization studies were carried out in strict accordance with the recommendations in the Guide for the Care and Use of Laboratory Animals of the German Federal Animal Protection Law. The protocols were approved by the Committee on the Ethics of Animal Experiments of the University of Ulm (Tierforschungszentrum Ulm, Oberberghof) and the Regierungspräsidium Tübingen (Permit Numbers 992, 1231, 1334, and 1384 to R.S.). All immunizations were performed under short time Isoflurane

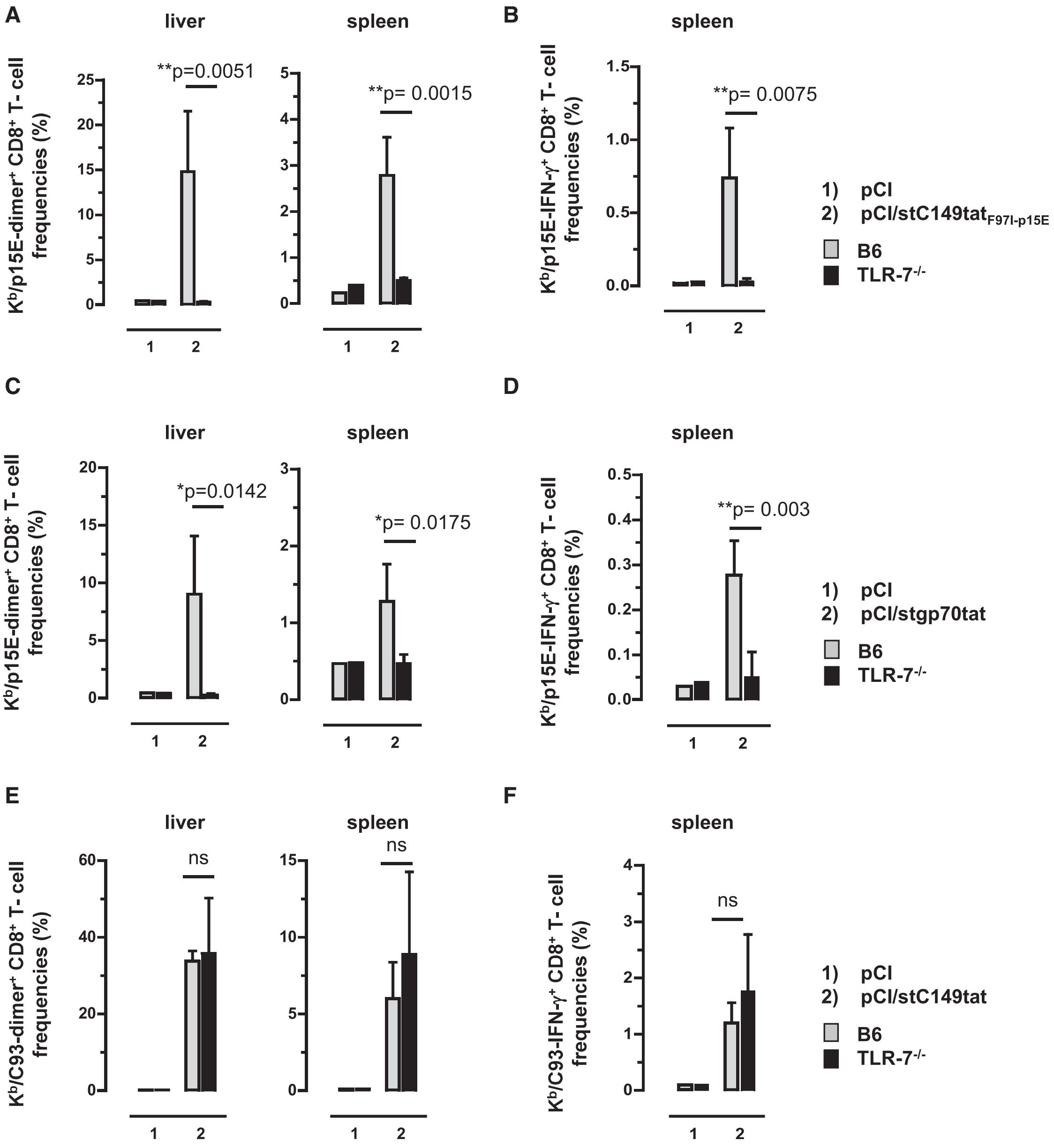

Figure 6. CD8+ T Cell Induction in Vaccinated B6 and TLR7$^{-/-}$ Mice

TLR7$^{-/-}$ and B6 mice were immunized intramuscularly (i.m.) (n = 3–4 mice/group) with empty pCI and pCI/stC149tat$_{F97I\text{-}p15E}$ (A and B), with empty pCI and pCI/stgp70tat (C and D), or with empty pCI and pCI/stC149tat (E and F). Twelve days post-injection, antigen-specific dimer+ CD8+ T cell responses were determined in the liver and spleen. Furthermore, spleen cells were *ex vivo* re-stimulated with K^b/C93 and K^b/p15E peptides or an irrelevant K^b/Ova257 peptide. Specific IFN-γ+ CD8+ T cell frequencies were determined by FCM, and K^b/Ova257-specific background values were subtracted. The mean percentages of dimer+ (A and C) and IFN-γ+ K^b/p15E-specific CD8+ T cells (B and D) in the corresponding CD8+ T cell populations (+SD) are shown. Furthermore, the mean percentages of dimer+ (E) and IFN-γ+ K^b/C93-specific CD8+ T cells (F) in the corresponding CD8+ T cell populations (+SD) are shown. The statistical significance of differences between vaccinated B6 and TLR7$^{-/-}$ mice was determined by the unpaired Student's t test. *$p < 0.05$ and **$p < 0.01$ were considered statistically significant. ns, not significant.

anesthesia, and all efforts were made to minimize suffering. BALB/cJ (BALB/c) and C57BL/6J (B6) (Janvier, France), TLR-7$^{-/-}$ (008380; Jackson), and 1.4HBV-S^{mut} tg mice[23] were bred and kept under standard pathogen-free conditions in the animal colony of Ulm University. Male 1.4HBV-S^{mut} tg mice were screened by analyzing blood samples with an Elecsys HBeAg immunoassay (COBAS cat. no. 11820583; Roche, Mannheim, Germany) and used in the immunization studies.

Plasmid Constructs

Antigen-encoding sequences were codon optimized and synthesized by GeneArt (Regensburg, Germany) or, where indicated, were modified from these constructs by PCR. PCRs were performed with the Q5 Site-Directed Mutagenesis Kit (cat. no. E05548; NEB, Frankfurt, Germany). All sequences were cloned into the pCI vector (cat. no. E1731; Promega, Mannheim, Germany). Batches of DNA were produced in *E. coli* using the QIAGEN Plasmid Mega Kit (cat. no. 12183; QIAGEN, Hilden, Germany).

Characterization of Antigen Expression in Transfected Cells

Human embryonic kidney cells (HEK293 cells; ATCC CRL-1573) were transiently transfected with the indicated plasmid DNAs using the calcium phosphate method. For western blot analyses, transiently transfected cells were directly lysed at 36–48 h post-transfection with SDS-containing buffer (62.5 mM Tris-hydrochloride; 3% SDS; supplemented with 5% mercaptoethanol [pH 6.8]), processed for SDS-PAGE, and blotted on Nitrocellulose membranes (cat. no. IB3010-01; Thermo Fisher, Germany) using the iBlot Dry Blotting system (Thermo Fisher). Nitrocellulose membranes were incubated for 6 h with polyclonal rabbit anti-core antiserum and/or mouse anti-β-actin monoclonal antibody (mAb; cat. no. A2228; Sigma, Munich, Germany), followed by a second (1-h) incubation with horseradish peroxidase (HRP)-labeled donkey anti-rabbit IgG (cat. no. NA934; GE Healthcare, Dornstadt, Germany) and/or sheep anti-mouse IgG (cat. no. NA931V; GE Healthcare, Dornstadt, Germany). Where indicated, membranes were incubated with Restore Western Blot Stripping Buffer (cat. no. 21059; Thermo Fisher Scientific) according to the recommendations of the manufacturer prior to incubation with the mouse anti-β-actin mAb. For detection of strep-tagged proteins, the strepMAB-classic (strep-tag II specific mAb) conjugated to HRP (cat. no. 2-1509-001; IBA Lifesciences, Göttingen, Germany) was used according to the manufacturer's instructions. The membranes were dried, and the HRP detection reagent was applied as recommended by the manufacturer (cat. no. WBKLS0100; Millipore, Darmstadt, Germany) followed by exposure to a radiography film (cat. no. 28906847; GE Healthcare, Dornstadt, Germany). Recombinant antigens were purified using the strep-tag purification system as described previously.[6]

Immunization of Mice

Mice were immunized into tibialis anterior muscles with 100 μg of plasmid DNA in PBS.

Determination of Antigen-Specific CD8$^+$ T Cell Frequencies

To determine IFN-γ-expressing CD8$^+$ T cell frequencies, we stimulated splenocytes (10^6/100 μL) *ex vivo* with antigen-specific (e.g., K^b/C93 or K^b/p15E) and control (e.g., K^b/OVA257-264) peptides (JPT, Berlin, Germany) for 4 h in Ultra Culture medium (cat. no. BE 12-725F; Lonza, Belgium) containing 5 μg/mL of the respective peptides and 0.5 μg/mL brefeldin A (cat. no. 15870; Sigma-Aldrich). Subsequently, cells were surface stained with allophycocyanin (APC)-conjugated anti-CD8 mAb (cat. no. 17-0081-83; eBioscience), fixed with 2% paraformaldehyde, resuspended in permeabilization buffer (HBSS, 0.5% BSA, 0.5% saponin, 0.05% sodium azide), and stained with phycoerythrin (PE)-conjugated anti-IFN-γ antibody (cat. no. 12-7311-82; eBioscience). Non-specific binding of antibodies to Fc-receptor was blocked by preincubating cells with mAb 2.4G2 (cat. no. 01241D; BD Biosciences, Heidelberg Germany) directed against the FcγRIII/II CD16/CD32 (0.5 μg mAb/10^6 cells/100 μL). Similarly, spleen cells were stained with APC-conjugated anti-CD8 mAb and PE-conjugated DimerX I:PE Soluble Dimeric Mouse H-2Kb:Ig Fusion Protein (cat. no. 552944; BD Biosciences, Heidelberg, Germany) loaded with peptides K^b/C93 or K^b/p15E or DimerX I:Recombinant Soluble Dimeric Mouse H-2Ld:Ig Fusion Protein (cat. no. 550751; BD Biosciences, Heidelberg, Germany) loaded with L^d/AH1 peptide, followed by staining with PE-conjugated anti-mouse-IgG1 antibody (cat. no. 550083; BD Biosciences, Heidelberg, Germany) for 30 min at 4°C. Frequencies of IFN-γ$^+$ CD8$^+$ T cells and dimer$^+$ CD8$^+$ T were determined by flow cytometry (FCM) using a BD LSR-II Flow Cytometer. In the described experiments, we analyzed 5–10 × 10^4 CD8$^+$ T cells and determined the actual percentage (%) of IFN-γ$^+$ or dimer$^+$ CD8$^+$ T cells. Analysis of dimer$^+$ CD8$^+$ T cell frequencies in the liver was performed as described previously.[24,25]

Quantification of HBV Replication in the Liver

Extraction of DNA from liver tissues was described previously.[24,25] Real-time qPCR was performed using the RT^2 SYBR Green ROX qPCR Mastermix (cat. no. 330529; QIAGEN, Hilden, Germany) according to the manufacturer's instructions with 50 ng of total genomic liver DNA and 400 nM HBV surface antigen-specific primers (forward 5′-GGG AAC TAC CGT GTG TCT TGG CC-3′; reverse 5′-AAC GCC GCA GAC ACA TCC AGC-3′) in 25-μL reactions. Normalization against housekeeping genes was performed with primers specific for β-actin (forward 5′-CTA CAA TGA GCT GCG TGT GGC C-3′; reverse 5′-GGC TGG GGT GTT GAA GGT CTC A-3′) or interleukin (IL)-20R2 (forward 5′-GGG ACA TTC CGG TGC ACC TAG AAA C-3′; reverse 5′-CCA CAC ACG TAG GCT GGC TGA AG-3′).[50] Cycling parameters were as follows: (I) 1 cycle: 95°C, 10 min; (II) 40 cycles: 95°C, 15 s, 60°C, 60 s; and (III) 1 cycle: 72°C, 2 min on a 7500 FAST Real-Time PCR System (Applied Biosystems, Foster City, CA, USA). The HBV surface antigen-encoding plasmid pCI/S was used as a standard and diluted with 0.05 ng/μL mouse genomic DNA of a wild-type B6 mouse to 10^{10}, 10^9, 10^8, 10^7, 10^6, 10^5, 10^4, 10^3, 10^2, 10^1, and 10^0 copies per reaction.

Characterization of gp70 mRNA Expression

RNA was isolated from cell culture cells or primary tissue with the QIAGEN RNeasy Mini Kit (cat. no. 74104; QIAGEN, Hilden, Germany) according to instructions of the manufacturer. cDNA synthesis was performed with iScript cDNA Synthesis Kit (cat. no. 1708890; Bio-Rad, Munich, Germany). gp70-specific PCRs were performed with the primer sets gp70/AKV (5′-CGA GCC AAA TAT AAA AGA GAA CCC-3′/5′-GGT GGT GAA CCA AGG GGA CTT-3′) and β-actin (5′-CAT GTT TGA GAC CTT CAA CAC CC-3′/5′-GCC ATC TCC TGC AAG TCT AG-3′) and additionally (623/7499 5′-GTA CGG GAT AGC ATG GCC AAA CTT AGA GAA-3′; 623/7694 5′-CTA CCG AAA TCC TGT CTT TGA TAA

ACT G-3′).[31] All primers were purchased from biomers.net (Ulm, Germany). Cycling parameters were as follows: (I) 1 cycle: 95°C, 10 min; (II) 30 cycles: 95°C, 10 s, 53°C, 30 s, 72°C, 1 min; and (III) 72°C, 10 min. PCR fragments were analyzed in 1.5% agarose gels.

In Vivo Killing Assay

Splenocytes from non-immunized mice were isolated and pulsed with specific K^b/p15E peptide (labeled with high CFSE) and/or irrelevant K^b/Ova257 peptide (labeled with low CFSE). A total of 2×10^7 cells in a volume of 200 μL (cell suspension 50% target cells and 50% irrelevant cells) were injected intravenously (i.v.) into mice at day 12 post-vaccination. Labeled cells were harvested 16 h post-transfer and analyzed by FCM. The CellTrace Proliferation Kit (cat. no. C34554; Invivogen, San Diego, CA, USA) was used as recommended by the manufacturer for CFSE labeling.

Statistics

PRISM 5.01 GraphPad software (GraphPad, San Diego, CA, USA) was used for statistical analyses. Figures show mean values + SD, and group sizes are stated in the figure descriptions. For the evaluation of statistical differences in the mean T cell frequencies between two groups, the unpaired Student's t test was used. For evaluation of statistical differences in the mean T cell frequencies, the HBV copy numbers between immunized 1.4HBV-S^{mut} tg mice and the statistical differences in tumor growth one-way ANOVA followed by Tukey's multiple comparisons (with 95% confidence intervals) were used. $^*p < 0.05$, $^{**}p < 0.01$, and $^{***}p < 0.001$ were considered significant.

SUPPLEMENTAL INFORMATION

Supplemental Information includes five figures and can be found online at https://doi.org/10.1016/j.ymthe.2019.01.011.

AUTHOR CONTRIBUTIONS

M.W., T.S., and R.S. conceived the experiments and secured funding. J.K., P.R., K.S., and G.R.-S. performed the experiments and analyzed the data. J.K. and R.S. wrote the manuscript. All authors edited and approved the manuscript.

CONFLICTS OF INTEREST

The authors declare no competing interests.

ACKNOWLEDGMENTS

We appreciate the expert technical assistance of Ellen Allmendinger and Katrin Schwengle. We thank Dr. Alexander Kleger (Internal Medicine I, University Hospital Ulm, Germany) for providing PDAC cell lines. This work was supported by grants (SCHI 505/5-1 and GRK-1789 'CEMMA') from Deutsche Forschungsgemeinschaft (DFG) and the International School in Molecular Medicine Ulm (to R.S.) and BIU Phase II TP I3 (Boehringer Ingelheim Ulm University BioCenter) (to M.W., R.S., and T.S.). J.K. is a PhD candidate at Ulm University. This work is submitted in partial fulfilment of the requirement for her PhD.

REFERENCES

1. Riedl, P., Stober, D., Oehninger, C., Melber, K., Reimann, J., and Schirmbeck, R. (2002). Priming Th1 immunity to viral core particles is facilitated by trace amounts of RNA bound to its arginine-rich domain. J. Immunol. *168*, 4951–4959.
2. Lee, B.O., Tucker, A., Frelin, L., Sallberg, M., Jones, J., Peters, C., Hughes, J., Whitacre, D., Darsow, B., Peterson, D.L., and Milich, D.R. (2009). Interaction of the hepatitis B core antigen and the innate immune system. J. Immunol. *182*, 6670–6681.
3. Fotin-Mleczek, M., Duchardt, K.M., Lorenz, C., Pfeiffer, R., Ojkić-Zrna, S., Probst, J., and Kallen, K.J. (2011). Messenger RNA-based vaccines with dual activity induce balanced TLR-7 dependent adaptive immune responses and provide antitumor activity. J. Immunother. *34*, 1–15.
4. Sominskaya, I., Skrastina, D., Petrovskis, I., Dishlers, A., Berza, I., Mihailova, M., Jansons, J., Akopjana, I., Stahovska, I., Dreilina, D., et al. (2013). A VLP library of C-terminally truncated Hepatitis B core proteins: correlation of RNA encapsidation with a Th1/Th2 switch in the immune responses of mice. PLoS ONE *8*, e75938.
5. Heidenreich, R., Jasny, E., Kowalczyk, A., Lutz, J., Probst, J., Baumhof, P., Scheel, B., Voss, S., Kallen, K.J., and Fotin-Mleczek, M. (2015). A novel RNA-based adjuvant combines strong immunostimulatory capacities with a favorable safety profile. Int. J. Cancer *137*, 372–384.
6. Krieger, J., Stifter, K., Riedl, P., and Schirmbeck, R. (2018). Cationic domains in particle-forming and assembly-deficient HBV core antigens capture mammalian RNA that stimulates Th1-biased antibody responses by DNA vaccination. Sci. Rep. *8*, 14660.
7. Riedl, P., Buschle, M., Reimann, J., and Schirmbeck, R. (2002). Binding immune-stimulating oligonucleotides to cationic peptides from viral core antigen enhances their potency as adjuvants. Eur. J. Immunol. *32*, 1709–1716.
8. Lau, C.M., Broughton, C., Tabor, A.S., Akira, S., Flavell, R.A., Mamula, M.J., Christensen, S.R., Shlomchik, M.J., Viglianti, G.A., Rifkin, I.R., and Marshak-Rothstein, A. (2005). RNA-associated autoantigens activate B cells by combined B cell antigen receptor/Toll-like receptor 7 engagement. J. Exp. Med. *202*, 1171–1177.
9. Kelly, K.M., Zhuang, H., Nacionales, D.C., Scumpia, P.O., Lyons, R., Akaogi, J., Lee, P., Williams, B., Yamamoto, M., Akira, S., et al. (2006). "Endogenous adjuvant" activity of the RNA components of lupus autoantigens Sm/RNP and Ro 60. Arthritis Rheum. *54*, 1557–1567.
10. Green, N.M., Moody, K.S., Debatis, M., and Marshak-Rothstein, A. (2012). Activation of autoreactive B cells by endogenous TLR7 and TLR3 RNA ligands. J. Biol. Chem. *287*, 39789–39799.
11. Bernard, J.J., Cowing-Zitron, C., Nakatsuji, T., Muehleisen, B., Muto, J., Borkowski, A.W., Martinez, L., Greidinger, E.L., Yu, B.D., and Gallo, R.L. (2012). Ultraviolet radiation damages self noncoding RNA and is detected by TLR3. Nat. Med. *18*, 1286–1290.
12. Roers, A., Hiller, B., and Hornung, V. (2016). Recognition of endogenous nucleic acids by the innate immune system. Immunity *44*, 739–754.
13. Rock, K.L., Hearn, A., Chen, C.J., and Shi, Y. (2005). Natural endogenous adjuvants. Springer Semin. Immunopathol. *26*, 231–246.
14. Saade, F., and Petrovsky, N. (2012). Technologies for enhanced efficacy of DNA vaccines. Expert Rev. Vaccines *11*, 189–209.
15. Li, L., and Petrovsky, N. (2017). Molecular adjuvants for DNA vaccines. Curr. Issues Mol. Biol. *22*, 17–40.
16. Wingfield, P.T., Stahl, S.J., Williams, R.W., and Steven, A.C. (1995). Hepatitis core antigen produced in Escherichia coli: subunit composition, conformational analysis, and in vitro capsid assembly. Biochemistry *34*, 4919–4932.
17. Ludgate, L., Liu, K., Luckenbaugh, L., Streck, N., Eng, S., Voitenleitner, C., Delaney, W.E., 4th, and Hu, J. (2016). Cell-free hepatitis B virus capsid assembly dependent on the core protein C-terminal domain and regulated by phosphorylation. J. Virol. *90*, 5830–5844.
18. Liao, W., and Ou, J.H. (1995). Phosphorylation and nuclear localization of the hepatitis B virus core protein: significance of serine in the three repeated SPRRR motifs. J. Virol. *69*, 1025–1029.
19. Selzer, L., Kant, R., Wang, J.C., Bothner, B., and Zlotnick, A. (2015). Hepatitis B virus core protein phosphorylation sites affect capsid stability and transient exposure of the C-terminal domain. J. Biol. Chem. *290*, 28584–28593.

20. Lin, Y.J., Huang, L.R., Yang, H.C., Tzeng, H.T., Hsu, P.N., Wu, H.L., Chen, P.J., and Chen, D.S. (2010). Hepatitis B virus core antigen determines viral persistence in a C57BL/6 mouse model. Proc. Natl. Acad. Sci. USA *107*, 9340–9345.

21. Yang, P.L., Althage, A., Chung, J., Maier, H., Wieland, S., Isogawa, M., and Chisari, F.V. (2010). Immune effectors required for hepatitis B virus clearance. Proc. Natl. Acad. Sci. USA *107*, 798–802.

22. Lin, Y.J., Wu, H.L., Chen, D.S., and Chen, P.J. (2012). Hepatitis B virus nucleocapsid but not free core antigen controls viral clearance in mice. J. Virol. *86*, 9266–9273.

23. Halverscheid, L., Mannes, N.K., Weth, R., Kleinschmidt, M., Schultz, U., Reifenberg, K., Schirmbeck, R., Nassal, M., Blum, H.E., Reimann, J., and Geissler, M. (2008). Transgenic mice replicating hepatitis B virus but lacking expression of the major HBsAg. J. Med. Virol. *80*, 583–590.

24. Riedl, P., Wieland, A., Lamberth, K., Buus, S., Lemonnier, F., Reifenberg, K., Reimann, J., and Schirmbeck, R. (2009). Elimination of immunodominant epitopes from multispecific DNA-based vaccines allows induction of CD8 T cells that have a striking antiviral potential. J. Immunol. *183*, 370–380.

25. Riedl, P., Reiser, M., Stifter, K., Krieger, J., and Schirmbeck, R. (2014). Differential presentation of endogenous and exogenous hepatitis B surface antigens influences priming of CD8(+) T cells in an epitope-specific manner. Eur. J. Immunol. *44*, 1981–1991.

26. Watanabe, T., Bertoletti, A., and Tanoto, T.A. (2010). PD-1/PD-L1 pathway and T-cell exhaustion in chronic hepatitis virus infection. J. Viral Hepat. *17*, 453–458.

27. Chan, H.W., Bryan, T., Moore, J.L., Staal, S.P., Rowe, W.P., and Martin, M.A. (1980). Identification of ecotropic proviral sequences in inbred mouse strains with a cloned subgenomic DNA fragment. Proc. Natl. Acad. Sci. USA *77*, 5779–5783.

28. White, H.D., Roeder, D.A., and Green, W.R. (1994). An immunodominant Kb-restricted peptide from the p15E transmembrane protein of endogenous ecotropic murine leukemia virus (MuLV) AKR623 that restores susceptibility of a tumor line to anti-AKR/Gross MuLV cytotoxic T lymphocytes. J. Virol. *68*, 897–904.

29. Huang, A.Y., Gulden, P.H., Woods, A.S., Thomas, M.C., Tong, C.D., Wang, W., Engelhard, V.H., Pasternack, G., Cotter, R., Hunt, D., et al. (1996). The immunodominant major histocompatibility complex class I-restricted antigen of a murine colon tumor derives from an endogenous retroviral gene product. Proc. Natl. Acad. Sci. USA *93*, 9730–9735.

30. Malarkannan, S., Serwold, T., Nguyen, V., Sherman, L.A., and Shastri, N. (1996). The mouse mammary tumor virus env gene is the source of a CD8+ T-cell-stimulating peptide presented by a major histocompatibility complex class I molecule in a murine thymoma. Proc. Natl. Acad. Sci. USA *93*, 13991–13996.

31. Kim, V., Yewdell, J.W., and Green, W.R. (2000). Naturally occurring TAP-dependent specific T-cell tolerance for a variant of an immunodominant retroviral cytotoxic T-lymphocyte epitope. J. Virol. *74*, 3924–3928.

32. Rammensee, H.G., Friede, T., and Stevanoviíc, S. (1995). MHC ligands and peptide motifs: first listing. Immunogenetics *41*, 178–228.

33. Takeda, J., Sato, Y., Kiyosawa, H., Mori, T., Yokoya, S., Irisawa, A., Miyata, M., Obara, K., Fujita, T., Suzuki, T., et al. (2000). Anti-tumor immunity against CT26 colon tumor in mice immunized with plasmid DNA encoding beta-galactosidase fused to an envelope protein of endogenous retrovirus. Cell. Immunol. *204*, 11–18.

34. Rice, J., Buchan, S., and Stevenson, F.K. (2002). Critical components of a DNA fusion vaccine able to induce protective cytotoxic T cells against a single epitope of a tumor antigen. J. Immunol. *169*, 3908–3913.

35. Schirmbeck, R., Riedl, P., Kupferschmitt, M., Wegenka, U., Hauser, H., Rice, J., Kröger, A., and Reimann, J. (2006). Priming protective CD8 T cell immunity by DNA vaccines encoding chimeric, stress protein-capturing tumor-associated antigen. J. Immunol. *177*, 1534–1542.

36. Dishlers, A., Skrastina, D., Renhofa, R., Petrovskis, I., Ose, V., Lieknina, I., Jansons, J., Pumpens, P., and Sominskaya, I. (2015). The hepatitis B virus core variants that expose foreign C-terminal insertions on the outer surface of virus-like particles. Mol. Biotechnol. *57*, 1038–1049.

37. McWilliams, J.A., Sullivan, R.T., Jordan, K.R., McMahan, R.H., Kemmler, C.B., McDuffie, M., and Slansky, J.E. (2008). Age-dependent tolerance to an endogenous tumor-associated antigen. Vaccine *26*, 1863–1873.

38. Gonzalez-Cao, M., Iduma, P., Karachaliou, N., Santarpia, M., Blanco, J., and Rosell, R. (2016). Human endogenous retroviruses and cancer. Cancer Biol. Med. *13*, 483–488.

39. Russell, R., Perkhofer, L., Liebau, S., Lin, Q., Lechel, A., Feld, F.M., Hessmann, E., Gaedcke, J., Güthle, M., Zenke, M., et al. (2015). Loss of ATM accelerates pancreatic cancer formation and epithelial-mesenchymal transition. Nat. Commun. *6*, 7677.

40. Schirmbeck, R., Kwissa, M., Fissolo, N., Elkholy, S., Riedl, P., and Reimann, J. (2002). Priming polyvalent immunity by DNA vaccines expressing chimeric antigens with a stress protein-capturing, viral J-domain. FASEB J. *16*, 1108–1110.

41. Wieland, A., Denzel, M., Schmidt, E., Kochanek, S., Kreppel, F., Reimann, J., and Schirmbeck, R. (2008). Recombinant complexes of antigen with stress proteins are potent CD8 T-cell-stimulating immunogens. J. Mol. Med. (Berl.) *86*, 1067–1079.

42. Kammerer, R., Stober, D., Riedl, P., Oehninger, C., Schirmbeck, R., and Reimann, J. (2002). Noncovalent association with stress protein facilitates cross-priming of CD8+ T cells to tumor cell antigens by dendritic cells. J. Immunol. *168*, 108–117.

43. Kurts, C., Robinson, B.W., and Knolle, P.A. (2010). Cross-priming in health and disease. Nat. Rev. Immunol. *10*, 403–414.

44. Liang, F., and Loré, K. (2016). Local innate immune responses in the vaccine adjuvant-injected muscle. Clin. Transl. Immunology *5*, e74.

45. Chintakuntlawar, A.V., Zhou, X., Rajaiya, J., and Chodosh, J. (2010). Viral capsid is a pathogen-associated molecular pattern in adenovirus keratitis. PLoS Pathog. *6*, e1000841.

46. Pertel, T., Hausmann, S., Morger, D., Züger, S., Guerra, J., Lascano, J., Reinhard, C., Santoni, F.A., Uchil, P.D., Chatel, L., et al. (2011). TRIM5 is an innate immune sensor for the retrovirus capsid lattice. Nature *472*, 361–365.

47. Crispe, I.N. (2014). Immune tolerance in liver disease. Hepatology *60*, 2109–2117.

48. Hong, M., and Bertoletti, A. (2017). Tolerance and immunity to pathogens in early life: insights from HBV infection. Semin. Immunopathol. *39*, 643–652.

49. Zamora, A.E., Crawford, J.C., and Thomas, P.G. (2018). Hitting the target: how T cells detect and eliminate tumors. J. Immunol. *200*, 392–399.

50. Wegenka, U.M., Dikopoulos, N., Reimann, J., Adler, G., and Wahl, C. (2007). The murine liver is a potential target organ for IL-19, IL-20 and IL-24: type I interferons and LPS regulate the expression of IL-20R2. J. Hepatol. *46*, 257–265.

YMTHE, Volume *27*

Supplemental Information

Endogenously Expressed Antigens Bind Mammalian RNA via Cationic Domains that Enhance Priming of Effector CD8 T Cells by DNA Vaccination

Jana Krieger, Petra Riedl, Katja Stifter, Gleyder Roman-Sosa, Thomas Seufferlein, Martin Wagner, and Reinhold Schirmbeck

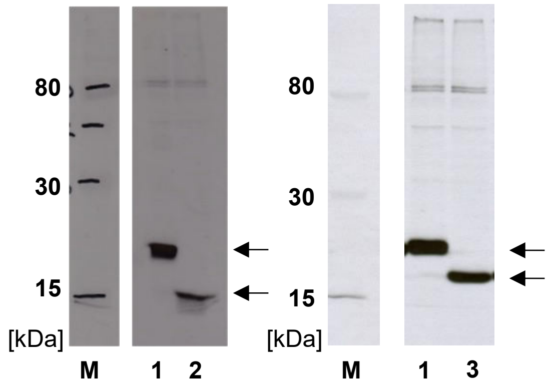

HEK-293 transfected with:

1) pCI/C
2) pCI/C149
3) pCI/C149tat

Supplementary Fig. S1

Expression of HBV-C, HBV-C149 and HBV-C149tat antigens in transiently transfected HEK-293 cells

Expression of the HBV core antigens was determined in two independent experiments. HEK-293 cells were transiently transfected with pCI/C (lanes 1), pCI/C149 (lane 2) or pCI/C149tat DNA (lane 3), labelled with 100µCi ^{35}S-methionine/cysteine at 36h post transfection for 12h and lysed with pH 8.0 lysis buffer (100 mM NaCl, 0.5% NP40 and 100 mM Tris-hydrochloride) supplemented with Protease Inhibitor Cocktail Tablets (cat. no. 11836145001, Roche Applied Science, Penzberg, Germany). Extracts were cleared by centrifugation and precipitated with a polyclonal rabbit anti core serum and protein-A sepharose. Precipitates were processed for SDS-PAGE and subsequent fluorography of the gels. The positions of the respective core antigens are indicated (black arrows). The molecular weight marker (in kDa) is indicated.

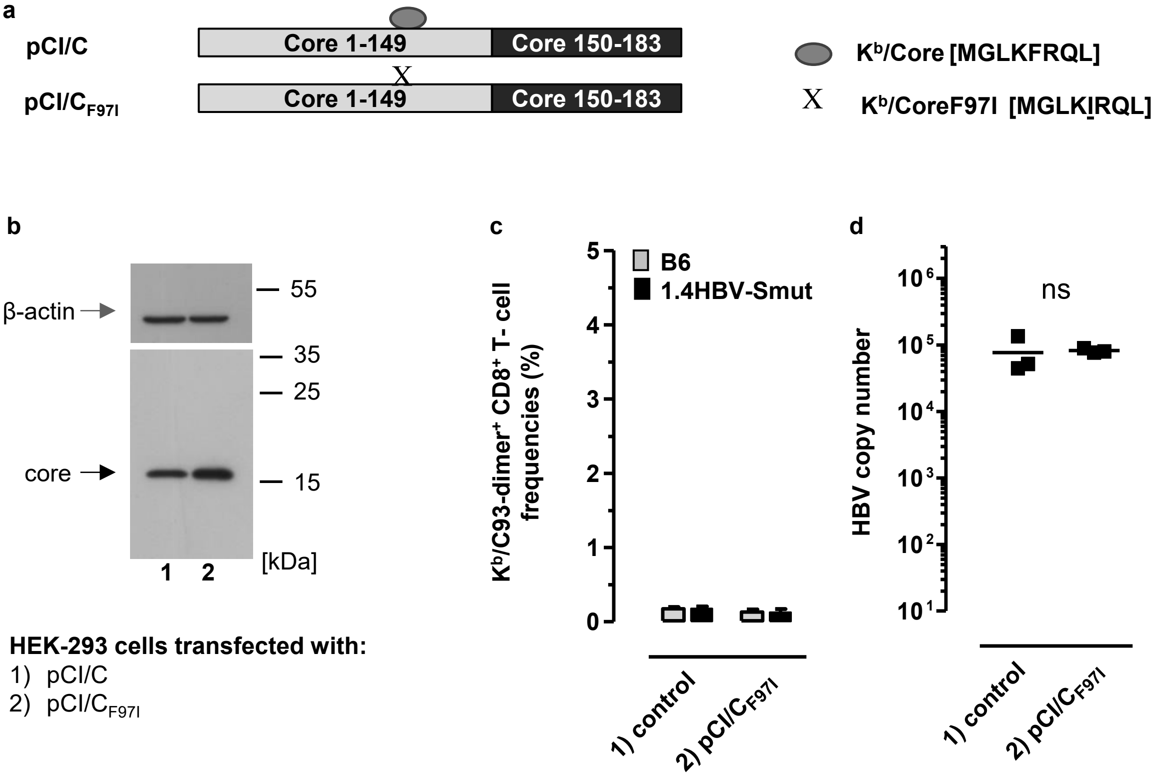

Supplementary Fig. S2

Characterization of the HBV-C antigen harbouring a mutated Kb/C93 epitope

(a) Schematic presentation of HBV-C and HBV-C_{F97I} antigens. The K^b/C93 epitope is given. The HBV-C_{F97I} construct harbors a point mutation at position C97 (exchange of an F to an I) and is indicated by a X. **(b)** Lysates of HEK-293 cells, transiently transfected with the indicated plasmids, were processed for SDS-PAGE followed by Core- and actin-specific western blot analysis. The positions of beta-actin (gray arrow) and HBV core antigens (black arrow) are shown. The molecular weight marker (in kDa) is indicated. **(c)** B6 and 1.4HBV-S^{mut} tg mice were either non-treated (control; groups 1; n=3) or injected with pCI/C_{F97I} (groups 2; n=3) DNA. Twelve days post immunization, K^b/C93-specific dimer$^+$ $CD8^+$ T-cell responses were determined in the liver. The mean % of K^b/C93-specific dimer$^+$ $CD8^+$ T cells in the hepatic $CD8^+$ T-cell populations ($\pm$SD) is shown. **(d)** HBV replication was determined in the liver of non-treated (control) and vaccinated 1.4HBV-S^{mut} tg mice by qRT-PCR as described in the M&M section. The statistical significance of differences between groups 1 and 2 was determined by the unpaired Student's t-test. ns: not significant.

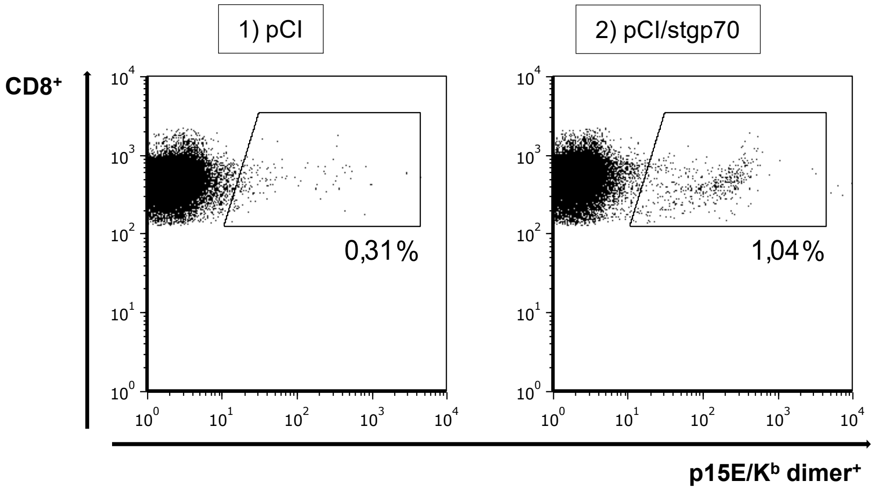

Supplementary Fig. S3

Representative FCM analysis of pCI and pCI/stgp70 vaccinated mice shown in figure 2c.

B6 mice were immunized i.m. with pCI (left panel) and pCI/stgp70 (right panel). Twelve days post immunization, antigen-specific K^b/p15E-dimer$^+$ CD8$^+$ T-cell responses were determined in preparations of spleens by FCM. In this experiment, two out of six pCI/stgp70-immune mice showed priming of K^b/p15E-dimer$^+$ CD8$^+$ T cells. One of the two positive K^b/p15E-dimer$^+$ CD8$^+$ T-cell populations are shown in dot blot analyses.

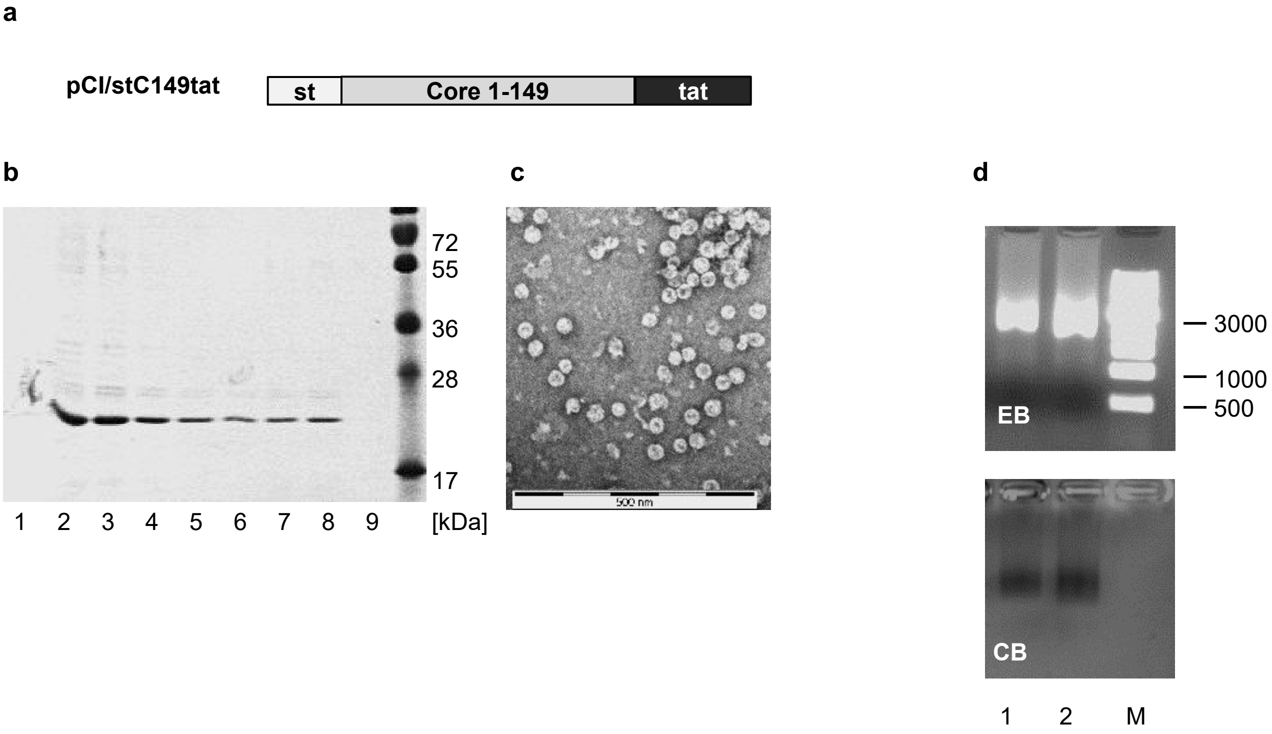

Supplementary Fig. S4

Characterization of HBV-stC149tat particles expressed in HEK-293 cells

(a) Schematic presentation of the HBV-stC149tat antigen. The strep-tag (st) and the HIV-tat domain are indicated. **(b)** HEK-293 cells were transiently transfected with pCI/stC149tat plasmid and recombinant protein was purified as described in M&M section. 10 µl of elution fractions (fractions 1 to 9) were processed for SDS-PAGE followed by Coomassie blue staining of the gel. The molecular weight marker is shown. (**c**) 2µg of purified protein was analysed on Joel TEM1400 at 100kV. The scale bar represents 500nm. (**d**) Two different HBV-stC149tat antigen preparations (1 and 2) were run on native agarose gels and stained with ethidium bromide (EB; upper panel) followed by Coomassie blue staining (CB; lower panel).

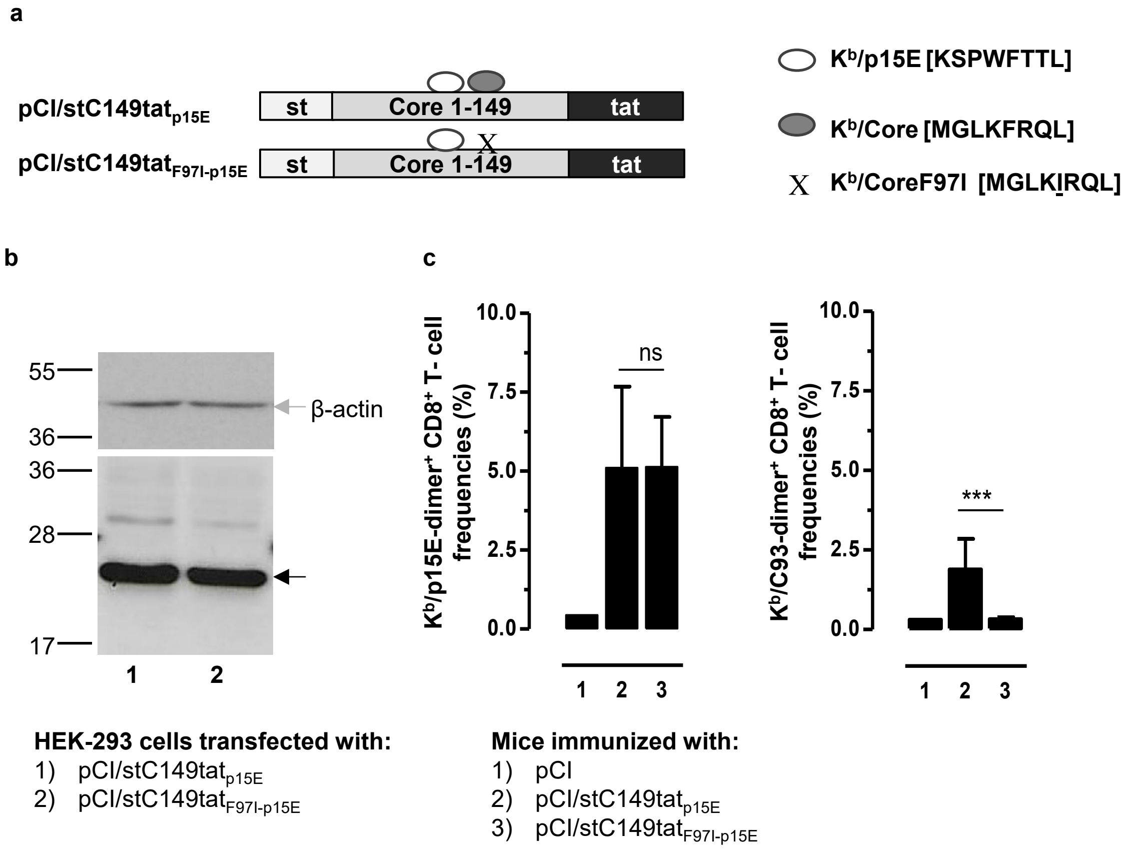

Supplementary Fig. S5

Induction of K^b/p15E-specific $CD8^+$ T-cell responses by DNA-based vaccines encoding chimeric HBV-C/p15E antigens

(a) Schematic presentation of pCI/stC149tat$_{p15E}$ and pCI/stC149tat$_{F97I\text{-}p15E}$ antigens. The chimeric pCI/stC149tat$_{F97I\text{-}p15E}$ antigen harboring the K^b/p15E and the silenced K^b/C93$_{F97I}$ epitopes is described in the result section and in legend to figure 2. Similarly, we constructed the pCI/stC149tat$_{p15E}$ vector encoding the chimeric HBV-stC149tat antigen with the K^b/p15E and the functional K^b/C93 epitopes. The K^b/p15E epitope, the K^b/C93 epitope and the mutant K^b/C93$_{F97I}$ amino acid sequence harboring the point mutation at position C97 (exchange of an F to an I) are indicated. **(b)** HEK-293 cells were transiently transfected with pCI/stC149tat$_{p15E}$ and pCI/stC149tat$_{F97I\text{-}p15E}$. Expression of the antigens was determined in cell lysates by western blot using strep-tag (black arrow) and beta-actin (gray arrow) specific antibodies. **(c)** B6 mice were immunized i.m. with pCI (group 1), pCI/stC149tat$_{p15E}$ (group 2; n=4) and pCI/stC149tat$_{F97I\text{-}p15E}$ (group 3; n=4). Twelve days post immunization, K^b/p15E-dimer$^+$ (left panel) and K^b/C93-dimer$^+$ (right panel) $CD8^+$ T-cell frequencies were determined in the spleen as described in the M&M section. The mean % of dimer$^+$ $CD8^+$ T cells in the splenic $CD8^+$ T-cell populations (+SD) are shown. The statistical significance of differences between groups 2 and 3 were determined by the unpaired Student's t-test. P values of $p < 0.001$ (***) were considered statistically significant. ns; not significant
